# Biomimetic cellular sponges for neutralizing inflammatory cytokines in osteoarthritic joints

**DOI:** 10.1016/j.mtbio.2026.103347

**Published:** 2026-06-11

**Authors:** Chenggong Ma, Zhisheng Xiao, Yetian Ma, Wenwei Jiang, Yufan Qian, Zicheng Deng, Jiong Jiong Guo, Qian Chen, Feng Zhou

**Affiliations:** a Department of Orthopaedic Surgery, The First Affiliated Hospital of Soochow University, Orthopedic Institute, Suzhou Medical College, Soochow University, Suzhou, Jiangsu, 215000, PR China; b Institute of Functional Nano and Soft Materials (FUNSOM), Jiangsu Key Laboratory for Carbon-Based Functional Materials and Devices, Soochow University, Suzhou, Jiangsu, 215123, PR China; c Department of Orthopaedic Surgery, Kunshan Hospital of Chinese Medicine, Affiliated Hospital of Yangzhou University, Jiangsu, 215300, PR China

**Keywords:** Osteoarthritis, Engineered macrophages membrane, HAMA hydrogel microspheres, Macrophage polarization, Cartilage organoid

## Abstract

Osteoarthritis (OA) is a joint disease characterized by age-related cartilage degradation, synovial inflammation, and imbalanced macrophage polarization. Pro-inflammatory cytokines, such as IL-1β and TNF-α, play critical roles in the progression of OA. Current treatments provide symptomatic relief but fail to address the underlying pathophysiological mechanisms, necessitating innovative therapeutic strategies. In this study, engineered macrophage membrane-incorporated hyaluronic acid methacrylate hydrogel microspheres (EMM@HMs) were used to neutralize pro-inflammatory cytokines and promote cartilage repair in osteoarthritic joints. The EMM@HMs were prepared using microfluidic techniques and characterized using scanning electron microscopy (SEM), zeta potential analysis, and particle size analysis. Cartilage organoids were prepared as a three-dimensional model to simulate native cartilage. The anti-inflammatory and cartilage-protective effects of the EMM@HMs were evaluated in vitro and in rats with OA. Results demonstrated that EMM@HMs neutralized IL-1β and TNF-α, promoted M2 macrophage polarization, and reduced cartilage degradation in cartilage organoids and OA rats. The EMM@HMs upregulated extracellular matrix (ECM)-related genes (COL2A1 and SOX9) and suppressed catabolic markers (MMP13 and COL10A1), highlighting their role in regulating ECM remodeling and chondrocyte differentiation. These findings demonstrated the potential of EMM@HMs to target inflammatory and degenerative pathways, offering a promising strategy for OA treatment.

## Highlights

• EMM@HMs act as “cellular sponges” that adsorb pro-inflammatory cytokines IL-1β and TNF-α.
• EMM@HMs promote M2 macrophage polarization, ameliorating the OA joint microenvironment.
• EMM@HMs achieve dual therapeutic effects of anti-inflammation and cartilage repair.

## Graphical abstract

RAW264.7 cells were transfected via lentivirus to overexpress IL1R2 and TNFR2, and the cell membranes were incorporated into HAMA microspheres using microfluidics to form EMM@HMs. Acting as cellular sponges, EMM@HMs could adsorb IL-1β and TNF-α in osteoarthritic joints, reducing inflammation and promoting M2 macrophage polarization. This shift improves the joint microenvironment, upregulates cartilage repair genes, and downregulates degradation markers, achieving dual effects of inflammation control and tissue repair in OA treatment.

**Figure:**
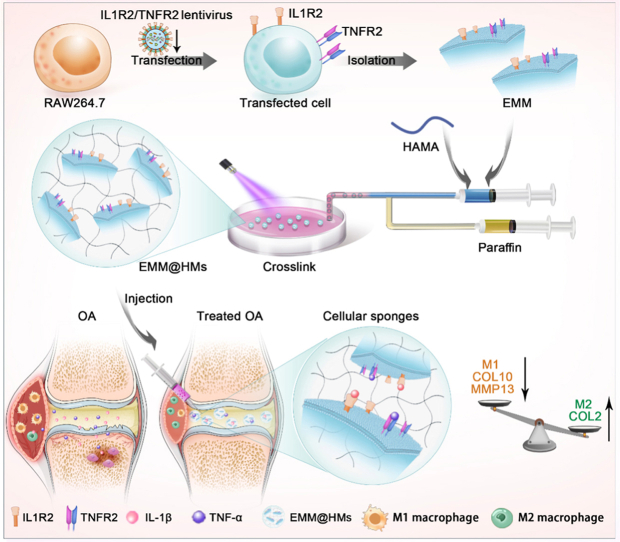

## 1 Introduction

Osteoarthritis (OA) is a common chronic joint disorder characterized by cartilage degradation, synovial inflammation, bone remodeling, ligament dysfunction, and osteophyte formation [1]. Clinically, OA manifests as joint pain, dysfunction, and deformities. The etiology of OA is complex and multifactorial and involves genetic predisposition, exercise-related injuries, aging, obesity, and sex differences [2]. Although OA has been extensively studied, its precise pathogenic mechanisms remain unclear [3,4]. OA can affect any joint, with the knees, hands, hips, and spine being the most affected. As one of the most prevalent orthopedic diseases worldwide, OA currently affects 7.6% (approximately 595 million) of the global population and is increasing [5]. This prevalence continues to increase, leading to substantial lower-limb disability and diminished quality of life. As the global population ages, the burden of OA escalates, creating a significant economic strain on both individuals and society. Therefore, the development of effective therapeutic strategies to address these public health challenges is of paramount importance.

Current treatments for OA include pharmacological therapy, physical therapy, surgical interventions, and lifestyle modifications. However, these treatments have notable limitations. Lifestyle modifications, including weight management and exercise, are preventive, but have limited efficacy in the advanced stages of OA. Physical therapy and rehabilitation can alleviate pain and improve joint function; however, their effectiveness varies among individuals and they cannot halt disease progression. Pharmacological treatments such as non-steroidal anti-inflammatory drugs (NSAIDs) and analgesics provide symptomatic relief but fail to reverse or halt joint damage [6]. Prolonged use of these drugs is associated with adverse side effects such as gastrointestinal bleeding. Surgical interventions can effectively alleviate pain and restore joint function; however, they involve higher risks and fail to fully replicate normal joint functionality [7,8]. Emerging therapies, such as stem cell therapy and platelet-rich plasma (PRP), show promise but remain experimental, with their clinical safety and efficacy yet to be validated [9,10]. These limitations highlight the urgent need for innovative strategies that not only alleviate symptoms but also target the underlying mechanisms of OA.

Paracrine interactions between macrophages and chondrocytes play a pivotal role in OA progression [11]. Infiltrating monocytes differentiate into M1 or M2 macrophages, which perform pro-inflammatory and tissue repair functions, respectively. Activated M1 macrophages secrete large amounts of cytokines, including IL-1β and TNF-α [12,13]. These cytokines are highly expressed in OA joints, exacerbating synovial inflammation and cartilage ECM degradation by inducing matrix metalloproteinases (MMPs) and promoting chondrocyte senescence. Additionally, M1 macrophages exhibit a highly active glycolytic state, leading to the accumulation of reactive oxygen species (ROS), which further drives the synthesis of inflammatory mediators and accelerates cartilage degradation [[14], [15], [16]]. In contrast, anti-inflammatory M2 macrophages play a critical role in tissue repair and resolution of inflammation [17,18]. Macrophages naturally express multiple receptors that enable them to absorb cytokines from the inflammatory microenvironment and promote resolution [19]. However, the expression levels of these receptors are relatively low, limiting their capacity to neutralize pro-inflammatory cytokines effectively and thus reducing their regulatory impact on inflammation. To enhance the therapeutic potential of macrophage membranes, engineered macrophage membranes (EMM) overexpressing TNF-α and IL-1β receptors have been developed [20]. These membranes more effectively bind and neutralize cytokines, reduce joint inflammation, and protect the cartilage from further damage.

Organoid technology has recently gained attention as a promising and innovative strategy to address the challenges of OA [19]. Organoids are three-dimensional (3D) microstructures derived from the directed differentiation of stem or progenitor cells with self-renewal and self-organizing capacities. They replicate key structural and functional properties of native tissues, offering extensive potential in disease modeling, drug testing, and regenerative medicine [21]. Cartilage organoids possess a remarkable ability to mimic the extracellular matrix composition and mechanical properties of native cartilage and closely replicate its structural and functional characteristics. Cartilage organoids support and promote cartilage repair and regeneration by providing biologically relevant environments. This innovative approach not only enhances our understanding of cartilage biology but also provides a valuable platform for modeling OA progression and testing therapeutic strategies [22].

**Scheme 1:**
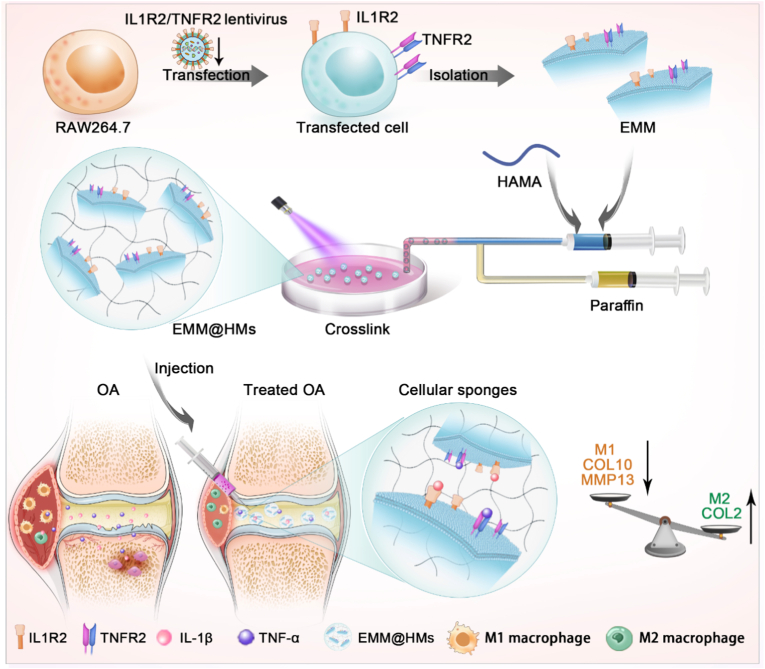
RAW264.7 cells were transfected using lentivirus to achieve high expression of IL1R2 and TNFR2 on their membranes. The engineered macrophage membranes were then isolated and incorporated into HMs using microfluidic technology to fabricate EMM@HMs. EMM@HMs function as “cellular nanosponges” in osteoarthritic joints, effectively adsorbing and neutralizing inflammatory factors, including IL-1β and TNF-α, thereby alleviating inflammation. Simultaneously, EMM@HMs promote macrophage polarization toward the M2 phenotype, optimizing the joint microenvironment. This process enhances the expression of cartilage repair-related genes (COL2 and ACAN) while reducing cartilage degradation markers (COL10 and MMP13). Ultimately, EMM@HMs exhibit dual functions in inflammation regulation and tissue repair, providing effective treatment for OA.

In this study, we developed an innovative therapeutic strategy by combining EMM with hyaluronic acid methacrylate (HAMA) hydrogel microspheres (EMM@HMs). The EMM neutralizes key pro-inflammatory cytokines, including TNF-α and IL-1β, alleviating joint inflammation. Simultaneously, the hydrogel provides a biocompatible scaffold for in vivo applications, whereas the cartilage organoids replicate native cartilage functions, supporting the repair and regeneration of damaged cartilage (Scheme 1). This study aimed to evaluate the efficacy and biocompatibility of EMM@HMs in an OA model, focusing on their ability to regulate macrophage polarization, inhibit chondrocyte hypertrophy, and neutralize key pro-inflammatory cytokines. By targeting the critical mechanisms underlying OA pathogenesis, EMM@HMs can sequester and neutralize inflammatory factors within the osteoarthritic joints. This dual action not only alleviates symptoms but also has the potential to modify OA progression, making it a groundbreaking therapeutic strategy for OA management.

## 2 Materials and methods

### 2.1 Reagents and media

RAW264.7 cells, a macrophage cell line, were cultured in Dulbecco's minimum essential medium (DMEM; Gibco, USA) supplemented with 10% fetal bovine serum (FBS; Gibco, USA), 100 U/mL penicillin (Gibco, USA), and 100 μg/mL streptomycin (Gibco, USA). ATDC5 cells, a chondrogenic cell line, were cultured in DMEM: nutrient mixture F12 (DMEM: F12; HyClone, USA) supplemented with 5% FBS, 100 U/mL penicillin, and 100 U/ml streptomycin. The cells were incubated at 37 °C under humidified conditions with 5% CO2. The bicinchoninic acid (BCA) Protein Assay Kit was purchased from Beyotime (China). Enzyme-linked immunosorbent assay (ELISA) reagents were obtained from MultiSciences (China). The Cell Count Kit-8 (CCK-8) reagent was purchased from Dojindo (Japan).

### 2.2 Preparation of knee joint specimens from OA patients

This clinical study was approved by the Ethics Committee of the First Affiliated Hospital of Soochow University (NO. 2023-134). Patients with primary knee OA diagnosed based on clinical symptoms and imaging findings who underwent total knee arthroplasty (TKA) were included in the study. Pre- and post-operative knee radiographs were collected, and patients were stratified using Kellgren-Lawrence (K-L) grading to assess disease severity. During TKA, specimens including the tibial plateau and synovial fluid were obtained. Tibial plateau cartilage samples were fixed in 4% paraformaldehyde for 48 h, decalcified in 10% EDTA for 6 weeks, embedded in paraffin, and sectioned at a thickness of 5 μm. Hematoxylin and eosin (H&E) and safranin O-fast green (S&F) staining were performed, and the degree of cartilage damage was evaluated using the Osteoarthritis Research Society International (OARSI) scoring system. Synovial fluid was stored at −80 °C for subsequent cytokine analysis.

### 2.3 ELISA analysis

ELISA was conducted to measure the expression of inflammatory factors, specifically IL-1β and TNF-α. Synovial-fluid samples were collected during TKA. For in vitro cell experiments, fluid samples were collected from Transwell co-culture chambers. In an animal study, plasma centrifuged from blood samples and synovial fluid from the knee joints of rats with OA were also obtained. To assess the expression levels of inflammatory factors IL-1β and TNF-α in synovial fluid and plasma, the supernatant was collected after centrifugation according to the manufacturer's instructions.

### 2.4 IL1R2/TNFR2 lentivirus preparation

IL1R2/TNFR2 shuttle plasmid vectors were constructed using GenePharma (China). Specifically, the prepared plasmids were transfected into HEK293T cells using Lipofectamine 3000 (Invitrogen, USA) for 48 h before filtration through a 0.45 μm cell filter. The supernatant was then centrifuged at 20,000 rpm for 120 min at 4 °C, followed by pellet resuspension and viral titer assays using qPCR.

### 2.5 Preparation and characterization of EMM@HMs

Cell membranes were collected following a previously described method [23]. Briefly, the cells were collected and washed thrice with PBS. They were then resuspended in a homogenization buffer containing 75 mM sucrose, 20 mM Tris-HCl, 2 mM MgCl_2_, 10 mM KCl, and protease/phosphatase inhibitors. The suspension was transferred to a Dounce homogenizer and disrupted by 20 passes to mechanically lyse the cells. The lysate was then centrifuged at 3200 × g for 5 min to remove large debris. The supernatant was collected and centrifuged at 20,000 g for 25 min to remove the pellet. The resulting supernatant was centrifuged at 100,000 × g for 35 min and the plasma membrane was collected as an off-white pellet. The extracted membranes were used for subsequent experiments, and the membrane protein concentration was quantified using a BCA kit. To prepare Did-labeled EMM@HMs (Did-EMM@HMs), the EMM was stained with Did dye (Beyotime Biotechnology, China) at a concentration of 10 μM. The microspheres were prepared using a coaxial needle microfluidic device with a 30 G inner needle and a 27 G outer needle. Briefly, HAMA (MW = 150 kDa), purchased from EFL-Tech (China), was dissolved in PBS at a concentration of 2% in the internal aqueous phase. The internal phase also contained 0.25% photoinitiator Lithium Phenyl (2,4,6-trimethyl benzoyl) phosphinate (LAP) and EMM. The outer oil phase was mineral oil with 3% Span 80 (Sigma-Aldrich, USA). The internal and outer phases were injected into the coaxial needle microfluidic device, with flow rates of 5 and 100 μL/min, respectively. Microsphere droplets were collected in a glass bottle and crosslinked by exposure to blue light at a wavelength of 405 nm for 15 s. The solidified microspheres were collected and washed with petroleum ether and isopropyl alcohol to remove the oil and surfactant. Finally, the microspheres were soaked in PBS and stored at 4 °C for further use. The morphology of the EMM@HMs was observed using bright-field microscopy, confocal microscopy, and scanning electron microscopy. The particle size was determined using a Malvern Mastersizer 2000 (UK), and the zeta potential was analyzed using a Zetasizer (Malvern Zetasizer Nano ZS90, UK).

### 2.6 SDS-PAGE assessment

Protein components of EMM@HMs were investigated by SDS-PAGE (GenScript, China). The total protein concentration was determined using a BCA kit. The SDS-PAGE assay was performed at 120 V for 90 min. The gel was then washed with TBST, stained with Coomassie Brilliant Blue, and decolorized to a clear and visible state. The gel with the protein bands was photographed. Molecular weight and purity were calculated using gel imaging system software.

### 2.7 Swelling and degradation rate tests

Swelling and degradation rate tests were conducted to characterize the biological performance of the EMM@HMs microspheres. To assess their swelling properties, fresh EMM@HMs microspheres were dried and weighed (W0). Ten milligrams of lyophilized HMs and EMM@HMs were placed in 1.5 mL EP tubes. Then, 1 mL of deionized water was added, and the tubes were incubated in a shaker (37 °C, 80 rpm). After 1, 3, 6, 9, and 12 h, the tubes were centrifuged at 5000 rpm for 5 min [24]. The supernatants were removed, and the HMs and EMM@HMs were weighed (Wt). The swelling rate was calculated as: Wt/W0. The degradation of the EMM@HMs microspheres was evaluated by analyzing their relative weight reduction. Specifically, 20 mg of HMs and EMM@HMs were immersed in 1 mL of PBS (pH 7.4) with 5 mg of hyaluronidase (Labgic Technology, China) and agitated at 37 °C and 80 rpm. The hyaluronidase solution was replaced every 2 days. At predetermined time points, the residual weight (Wt) of each sample was measured and compared with its initial weight (W0) [25]. The degradation rate was calculated as Wt/W0.

### 2.8 Cell biocompatibility

Cell biocompatibility and cytotoxicity were assessed using the CCK-8 assay and flow cytometry, respectively, in a Transwell system. ATDC5 cells were incubated in 24-well plates at 5 × 10^4^/well and co-cultured with HMs, EMM, and EMM@HMs for various time points. For the CCK-8 assay, 10% CCK-8 solution was added to each well at the predetermined evaluation time point, and the cells were incubated at 37 °C for 2 h. Absorbance was measured at 450 nm using a microplate reader. For flow cytometry, ATDC5 cells were collected, washed, resuspended, and then stained with 5 μL Annexin V-FITC and 5 μL propidium iodide (PI) for 15 min at 37 °C in the dark. The cells were then resuspended and diluted to appropriate concentrations. Apoptosis was evaluated and visualized by flow cytometry.

### 2.9 Construction of cartilage organoids

Cartilage organoids were constructed using specialized organoid culture platforms (Jiyan Biotech, China). Organoid culture inserts were prepared by reducing adhesion and removing bubbles, according to the manufacturer's instructions. ATDC5 cells were then seeded at 5 × 10^4^ cells/well into the inserts containing micropores at the bottom for cultivating three-dimensional chondrocyte clusters. The cells were incubated at 37 °C for 48 h. Finally, cartilage organoids were collected for further studies. To induce the chondrogenic differentiation of ATDC5 cells, 10 μg/mL Insulin-Transferrin-Selenium (ITS) was added to the culture medium.

### 2.10 Macrophage polarization induction and M1 conditioned medium (M1-CM) collection

RAW264.7 macrophages were cultured at 37 °C overnight and then stimulated with 100 ng/ml LPS and 20 ng/ml IFN-γ for 24 h to induce M1 phenotype polarization. The supernatant of the induced M1 macrophages was collected, centrifuged at 1000×g for 5 min, and diluted with serum-free medium (1:1) to obtain M1-CM. According to the protocol, M1-CM and different drugs were co-cultured with cartilage organoids in transwell chambers for further in vitro investigation. Immunofluorescence and flow cytometry were used to study the polarization of macrophage phenotypes. CD86 was chosen as the marker for M1 macrophages and CD206 for M2 macrophages.

### 2.11 qPCR analysis

For qPCR analysis, cartilage organoids were washed with PBS and lysed with TRIzol reagent (Invitrogen, USA) to obtain total RNA. TaqMan reverse transcription reagent (Applied Biosystems, USA) was used to synthesize cDNA. qPCR was performed using an ABI 7500 system (Applied Biosystems, USA) to detect the mRNA expression levels of COL2A1, ACAN, SOX9, COL1A1, COL10A1, and RUNX2. The primers used for the PCR are listed in Table S1. Data were analyzed using the 2^−ΔΔCt^ method and were normalized to β-actin. The gene-specific primers used for qPCR analysis are listed in the supplement table.

### 2.12 Western blot analysis

Cartilage organoids were lysed with RIPA buffer (Millipore, USA) to obtain total protein. The protein concentration was determined using a BCA kit. The proteins were separated using SDS-PAGE, then transferred to polyvinylidene fluoride (PVDF) membranes, and blocked with 5% nonfat milk for 2 h. Membranes were then incubated overnight at 4 °C with different primary antibodies, according to the manufacturer's instructions. After secondary antibody incubation and washing with TBST, bands were developed using ECL and visualized.

### 2.13 Immunofluorescence staining

For immunofluorescence staining, the cartilage organoids were fixed with 4% paraformaldehyde for 15 min and permeabilized with 0.1% Triton X-100 for 10 min. Organoids were blocked with 1% BSA for 1 h and then incubated with specific target antibodies. DAPI was used to stain the nucleus, and phalloidin was used to stain the cytoskeleton. Images were acquired using a confocal fluorescence microscope (Leica TCS-SP5, Germany).

### 2.14 Rat model of OA

Ethical approval for animal experiments was obtained from the Committee of the First Affiliated Hospital of Soochow University (NO. 2024-106). Thirty 8-week-old male Sprague-Dawley (SD) rats were provided by the Laboratory Animal Center of Soochow University and were divided equally into five groups (NC, OA, OA + HMs, OA + EMM, and OA + EMM@HMs). The rats in the NC group underwent a sham operation and were intra-articularly injected with PBS. Anterior cruciate ligament transection (ACLT) combined with medial meniscus transection (MMT) surgeries were performed on the right knee joints of rats in the other groups. Briefly, the rats were anesthetized, and the fur in the knee joint operation area was removed using a shaver. After disinfection, the anterior cruciate ligament and medial meniscus were gently destroyed using microscissors. The surgical area was rinsed with PBS, and the wound was sutured. Starting from the fourth week after surgical modeling, 30 μL of PBS, HMs, EMM, and EMM@HMs was injected weekly into the knee joint. EMM@HMs were prepared at a concentration of 10 mg/mL [25]. The rats were sacrificed 8 weeks after surgery. Blood samples and major organs, including the heart, liver, spleen, lungs, and kidneys, were collected.

### 2.15 Radiographic evaluation

For the in vivo radiological examination, radiography and micro-CT were performed to observe imaging changes in the knee joints with different treatments. Anterior and lateral radiographic imaging of the joints was performed to assess the degree of joint stenosis and the efficacy of the different treatments. Micro-CT (Skyscan1176, USA) was used to visualize the knee joint specimens of the rats. Briefly, the rats were sacrificed, and their knee joints were removed and treated with 4% paraformaldehyde for 48 h. Scan parameters were set to a 70 kV scanning voltage and a 10 μm scan thickness. Relevant parameters, including the total osteophyte volume, bone volume fraction (BV/TV), trabecular thickness (Tb.Th), and trabecular separation (Tb.Sp) were calculated and analyzed.

### 2.16 Histologic and immunohistochemical examinations

Eight weeks post-surgery, the rats were sacrificed, and knee joint specimens were collected. The samples were fixed for 48 h, decalcified for 6 weeks, and embedded in paraffin. Five-micrometer cryosections across the medial compartment of the knee joints in the sagittal position were prepared and stained with H&E and S&F. The OARSI scoring system was used to assess chondrocyte degeneration, and the Krenn score was applied to evaluate synovitis. Scoring criteria were based on previous studies. [26,27]. H&E staining of major organs, including the heart, liver, spleen, lungs, and kidneys, was also performed. Immunohistochemical staining was performed using primary antibodies according to the manufacturer's instructions. Immunohistochemical signals were imaged using a light microscope, and data analysis was performed using ImageJ software.

### 2.17 RNA-seq analysis

The function of EMM@HMs in vivo was analyzed by transcriptome analysis conducted by OE Biotech Co., Ltd. (China) using the OE Cloud Analysis Platform. Total RNA was isolated using TRIzol and quantified using a NanoDrop ND-100 (Thermo Fisher Scientific, USA). A sequencing library was constructed using the total RNA from all samples. After mRNA isolation and fragmentation, reverse transcription was performed to synthesize double-stranded cDNA. After terminal repair, connection, amplification, and purification, the quality of the library was inspected before sequencing, using an Illumina HiSeq4000 sequencer (Illumina, USA) to generate raw reads. Clean reads were obtained using FASTP and aligned with the reference genome using HISAT2. Principal component analysis (PCA) was performed using R software. Differentially expressed genes (DEGs) were identified by differential expression analysis using the DESeq2 R package. Genes with a q-value <0.05 and absolute fold change >2.0 were considered DEGs. Gene Ontology (GO), Kyoto Encyclopedia of Genes and Genomes (KEGG), Reactome, and Gene Set Enrichment Analysis (GSEA) of DEGs were conducted using the clusterProfiler R package.

### 2.18 Statistical analysis

All data are expressed as mean ± standard deviation (SD). Normality of the data was assessed using the Shapiro-Wilk test. For normally distributed data, differences between two groups were analyzed using a two-tailed Student's t-test, whereas comparisons among multiple groups were performed using one-way analysis of variance (ANOVA). For non-normally distributed data, the Wilcoxon rank-sum test or Kruskal-Wallis (K-W) test was applied as appropriate. ∗p < 0.05 and ∗∗p < 0.01 were considered statistically significant. Data analysis was performed using the SPSS software (version 22.0; SPSS Inc., USA).

## 3 Results

### 3.1 Synovial fluid inflammatory factors associated with cartilage degeneration in OA patients

**Fig. 1:**
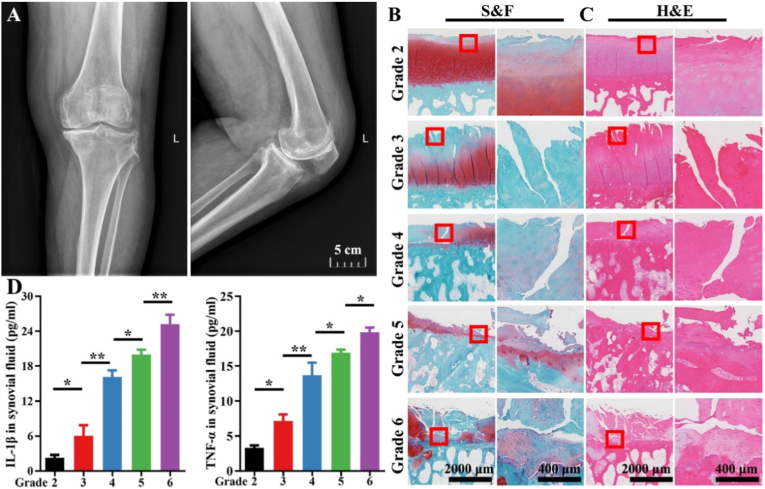
Correlation between histological assessment and cytokine levels in synovial fluid. (A) Radiographic analysis showed the reduced medial joint space in a patient with medial compartment OA. (B) H&E and (C) S&F staining illustrated cartilage degeneration in OA. (D) ELISA analysis of synovial fluid indicated the elevated TNF-α and IL-1β levels with OA progression. n = 5. ∗p < 0.05, ∗∗p < 0.01.

Analysis of the anterior and lateral radiographic views demonstrated that the patient exhibited medial compartment OA, as evidenced by a significantly narrower medial joint space compared to the lateral side (Fig. 1A). Postoperative anterior and lateral radiographs of the knee joint following TKA were also obtained (Fig. S1A). The tibial plateau and synovial fluid were collected during TKA. Severe wear of the articular cartilage on the medial tibial plateau was observed, in contrast to that on the lateral side. The central region of the medial tibial plateau is demarcated using a red box (Fig. S1B).

Decalcified articular cartilage sections were stained with H&E and S&F (Fig. 1B and C). The OARSI scoring system was used to evaluate the extent of cartilage damage. Additionally, the concentrations of TNF-α and IL-1β in synovial fluid were quantified through ELISA, revealing a progressive increase in their levels correlating with the advancement of OA (Fig. 1D). These findings indicate that elevated levels of synovial cytokines, including IL-1β and TNF-α, are associated with cartilage degeneration in OA.

### 3.2 Synthesis and characterization of EMM@HMs

**Fig. 2:**
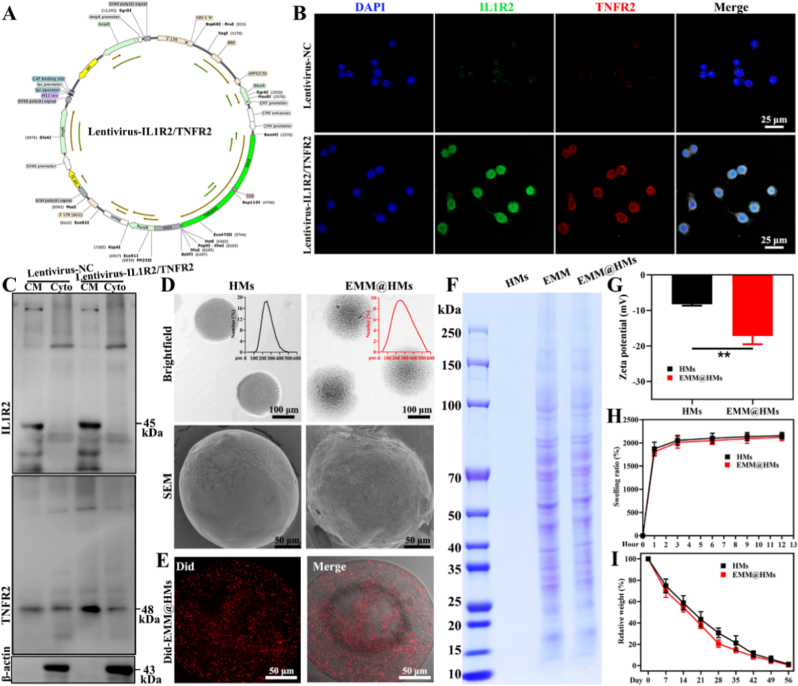
Design and characterization of EMM@HMs. (A) Schematic representation of the IL1R2/TNFR2 lentiviral vector construction. (B) Immunofluorescence showed IL1R2 and TNFR2 expression in RAW264.7 macrophages post-lentiviral transfection. n = 4. (C) Western blot analysis confirmed IL1R2 and TNFR2 protein expression, n = 3. (D) Bright-field and SEM images of HMs and EMM@HMs. CM indicates the cell membrane, n = 3. (E) Confocal microscopy images showed Did-EMM@HMs. n = 3. (F) SDS-PAGE analysis verified successful cell membrane encapsulation. n = 4. (G) Zeta potential analysis demonstrated surface charge changes in EMM@HMs. n = 3. (H) Swelling properties and (I) degradation characteristics of the microspheres. n = 4. ∗∗p < 0.01.

We constructed a lentiviral vector to induce the overexpression of IL1R2 and TNFR2, two key receptors for IL-1β and TNF-α, respectively (Fig. 2A). The lentivirus was then transfected into RAW264.7 macrophages. Immunofluorescence staining demonstrated increased IL1R2 and TNFR2 signals in the Lentivirus-IL1R2/TNFR2 group compared with the Lentivirus-NC group (Fig. 2B), and the corresponding fluorescence intensity was significantly elevated, as shown by quantitative analysis (Fig. S2A). qPCR further confirmed that IL1R2 and TNFR2 mRNA levels were markedly upregulated after lentiviral transfection (Fig. S2B). In addition, western blot analysis confirmed increased protein expression of IL1R2 and TNFR2 (Fig. 2C). This increase was especially pronounced in the cell membrane, indicating successful receptor overexpression on the macrophage membrane (Fig. S2C). Together, these results confirmed the successful construction and transfection of the IL1R2/TNFR2 lentiviral system in RAW264.7 macrophages.

Bright-field and SEM showed that the HMs were transparent with a smooth surface, whereas the EMM@HMs appeared opaque owing to the encapsulation of cell membranes (Fig. 2D). To verify this, the cell membrane was labeled with Did, a lipophilic fluorescent dye used to stain cell membranes. Laser confocal microscopy revealed the localization of Did-EMM in HMs (Fig. 2E). Finally, the EMM@HMs were dissolved and analyzed by electrophoresis, followed by Coomassie Brilliant Blue staining (Fig. 2F). These results confirmed that the membrane proteins were successfully encapsulated in the microspheres.

HMs exhibited a zeta potential of approximately −8 mV, while EMM@HMs showed a reduced zeta potential of approximately −17 mV (Fig. 2G). We further evaluated the swelling ratio of the microspheres and observed that both the HMs and EMM@HMs demonstrated excellent water absorption capabilities, with their swelling ratios stabilizing after 3 h (Fig. 2H). We then investigated the degradation behavior of the microspheres and found that both the HMs and EMM@HMs exhibited similar mass loss trends during degradation (Fig. 2I). This result indicated that the loaded materials did not significantly affect the swelling ratio or degradation characteristics of the microspheres.

### 3.3 EMM@HMs inhibit M1-CM-induced cartilage organoid hypertrophy

**Fig. 3:**
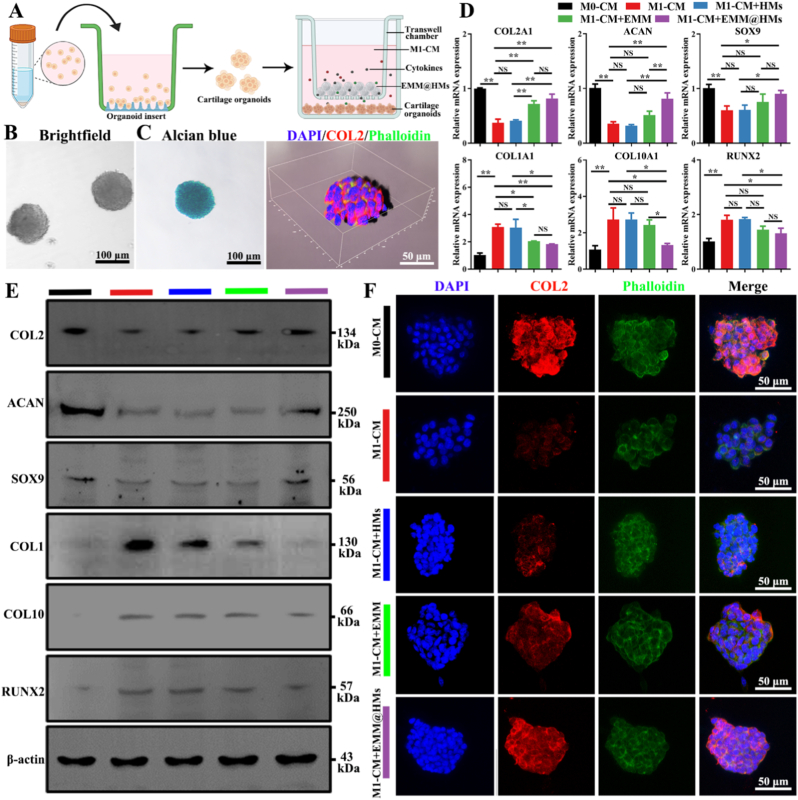
EMM@HMs protect cartilage organoids from hypertrophy under OA conditions. (A) Schematic representation of cartilage organoid construction and the co-culture system. (B) Bright-field microscopy showed uniform morphology of the cartilage organoids. n = 4. (C) Alcian blue and immunofluorescence staining of COL2 of cartilage organoids. n = 4. (D) qPCR analysis of cartilage matrix synthesis and degradation markers. n = 4. (E) Western blot analysis of protein expression levels. (F) Immunofluorescence images demonstrated cartilage matrix markers. n = 3. ∗p < 0.05, ∗∗p < 0.01, NS means not significant.

We established a cartilage organoid model to investigate the regulatory effects of different materials under OA conditions (Fig. 3A). The cartilage organoids were generated using a three-dimensional culture system. Bright-field microscopy showed that the organoids exhibited a uniform spherical morphology (Fig. 3B), which was distinct from the typical monolayer morphology of ATDC5 cells cultured in conventional plates (Fig. S3A and B). In addition, Alcian blue staining and immunofluorescence staining confirmed the cartilage-like phenotype of the organoids, as evidenced by the expression of the cartilage markers COL2 and ACAN (Fig. 3C and S3C). AFM analysis further revealed the surface topography and mechanical properties of the cartilage organoids, supporting their cartilage-like characteristics (Fig. S3D). The in vitro biosafety of EMM@HMs was assessed by CCK-8 assay and flow cytometry. The results showed no significant differences in cell proliferation between the groups at 24, 48, and 72 h (Fig. S4A). The flow cytometry results indicated consistently low apoptosis rates, with no significant differences across all groups (Fig. S4B and C). Subsequently, we analyzed the expression of cartilage matrix synthesis and degradation markers using qPCR, western blot, and immunofluorescence. qPCR results revealed that compared with M0 conditioned medium (M0-CM)-treated group, the M1-CM treatment group suppressed the expression of cartilage marker genes, such as COL2A1, ACAN, and SOX9, while promoting the expression of cartilage degradation-related genes, including COL1A1, COL10A1, and RUNX2. However, the addition of EMM@HMs restored the expression of the cartilage marker genes (Fig. 3D). Western blot analysis further confirmed the corresponding changes at the protein level (Fig. 3E), and the quantitative results are shown in Fig. S3E. In addition, immunofluorescence staining demonstrated the protective effects of EMM@HMs on cartilage matrix markers (Fig. 3F), with quantitative analysis of COL2 fluorescence intensity presented in Fig. S3F. These findings indicate that EMM@HMs preserve the cartilage-like phenotype of organoids under OA conditions by promoting matrix synthesis and attenuating degenerative changes.

### 3.4 EMM@HMs regulate macrophage polarization

**Fig. 4:**
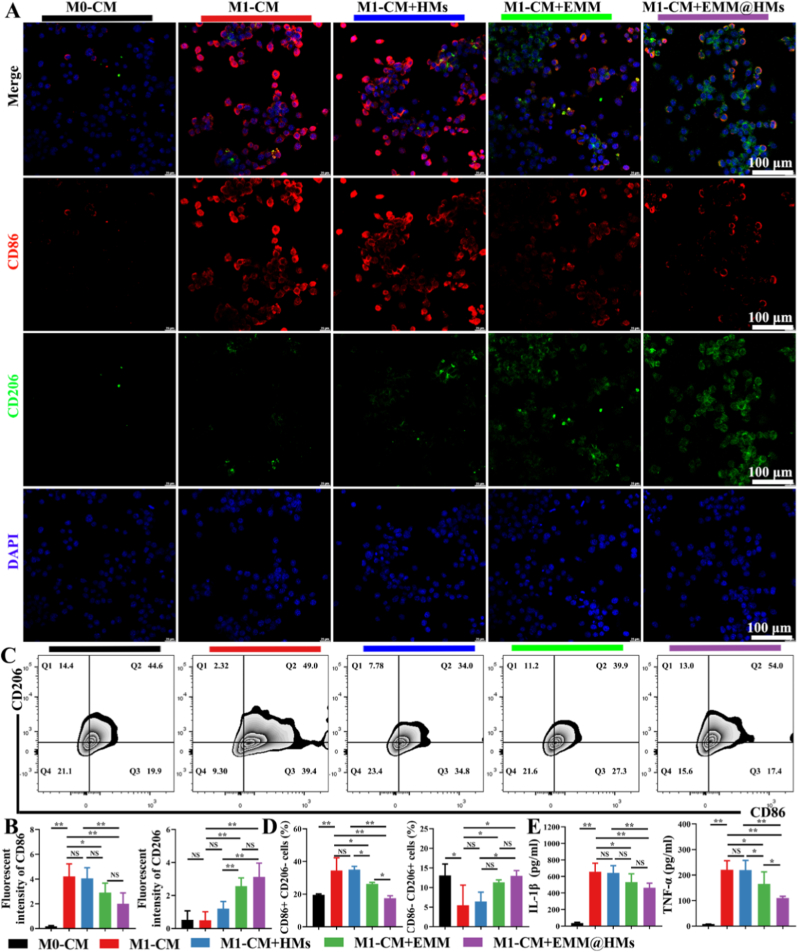
Regulation of macrophage polarization by EMM@HMs. (A) Immunofluorescence showed EMM@HMs reduced CD86 and increased CD206 expression. n = 3. (B) Quantitative analysis confirmed a significant shift toward M2 polarization in the EMM@HMs group. n = 3. (C) Flow cytometry demonstrated distinct macrophage polarization profiles under the treatments of EMM@HMs. n = 3. (D) Statistical analysis showed EMM@HMs decreased M1 and increased M2 macrophage percentages. n = 3. (E) ELISA results revealed EMM@HMs reduced pro-inflammatory cytokines IL-1β and TNF-α. n = 4. ∗p < 0.05, ∗∗p < 0.01, NS means not significant.

We examined the effects of different materials on macrophage polarization. Immunofluorescence staining revealed that M1-CM treatment increased CD86 expression and reduced CD206 expression, indicating enhanced M1 polarization. By contrast, EMM@HMs counteracted this trend by reducing CD86 expression and enhancing CD206 expression, thereby promoting M2 polarization (Fig. 4A and B). Flow cytometry analysis revealed that M1-CM treatment increased the proportion of M1 macrophages and reduced the number of M2 macrophages. By contrast, the EMM@HMs reversed these changes (Fig. 4C and D). To further validate these findings, we measured macrophage polarization-related cytokines, IL-1β and TNF-α, using ELISA. M1-CM treatment elevated IL-1β and TNF-α levels. Conversely, the EMM@HMs treatment reduced the levels of these cytokines (Fig. 4E). These findings indicated that M1-CM drives M1 macrophage polarization, whereas EMM@HMs counteract this effect and facilitate M2 polarization.

### 3.5 EMM@HMs delayed cartilage degeneration and alleviated synovitis

First, we evaluated the intra-articular retention time of EMM@HMs. In vivo imaging showed no significant difference in fluorescence intensity between Did-EMM and Did-EMM@HMs at baseline (Fig. S5A and B). Following intra-articular injection into rat knee joints, Did-EMM exhibited detectable fluorescence signals for up to 3 days, whereas Did-EMM@HMs remained detectable throughout the 14-day observation period (Fig. S5C and D). These results indicated that EMM@HMs markedly prolonged the intra-articular retention of EMM. Notably, day 14 was the last time point at which fluorescence signals remained detectable in this study, rather than the complete clearance endpoint.

**Fig. 5:**
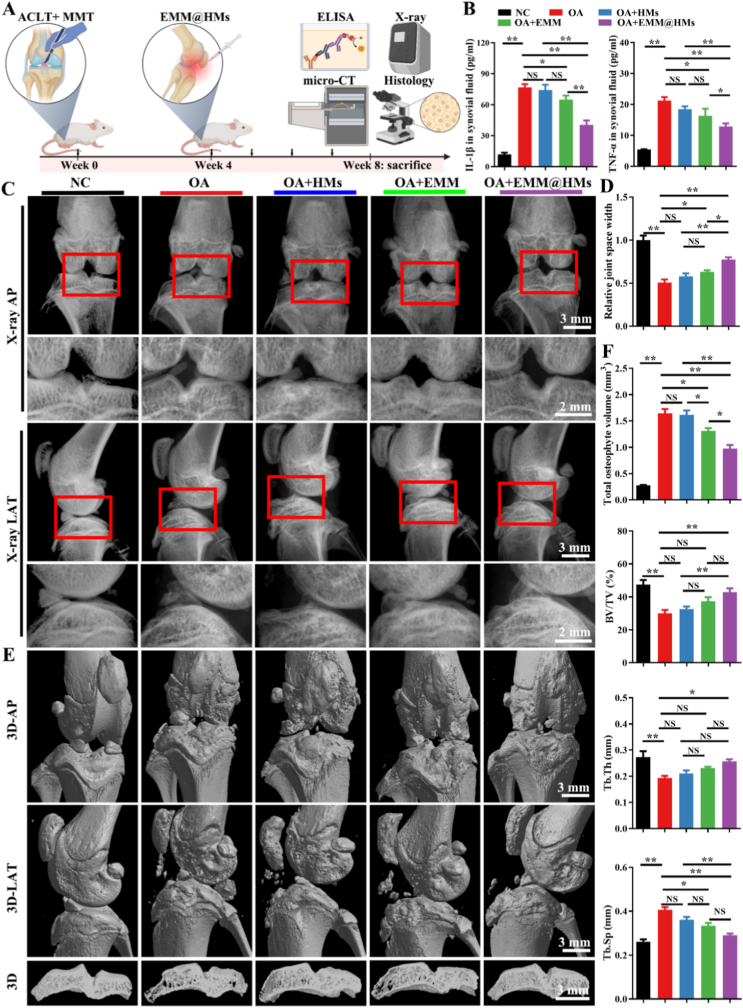
Protective effects of EMM@HMs on joint structure and inflammation in an OA rat model. (A) A flowchart illustrated the induction of OA in rats, intra-articular treatments, and subsequent sample analysis. (B) EMM@HMs reduced inflammatory cytokines IL-1β and TNF-α levels in synovial fluid. n = 4. (C) EMM@HMs restored joint space and reduced osteophyte formation, as shown by X-ray imaging. n = 4. (D) Quantitative analysis showed that EMM@HMs improved joint space width in X-ray images. n = 4. (E) Effects of EMM@HMs on revealing osteophyte cartilage damage and bone deterioration assessed by micro-CT. n = 4. (F) Quantitative micro-CT analysis. n = 4. ∗p < 0.05, ∗∗p < 0.01, NS means not significant.

Furthermore, we established an OA model by performing ACLT combined with MMT, then treated with HMs, EMM, and EMM@HMs (Fig. 5A). ELISA results showed elevated IL-1β and TNF-α levels in OA synovial fluid and plasma compared to the NC group, indicating severe inflammation. Treatment with HMs, EMM, or EMM@HMs reduced the levels of these cytokines, with EMM@HMs showing the strongest anti-inflammatory effects (Fig. 5B and S6). We further evaluated structural changes in the joints using X-ray imaging and micro-CT. Radiographic analysis revealed a severe narrowing of the joint space in the OA group. By contrast, treatment with EMM and EMM@HMs restored the joint space width (Fig. 5C and D). Micro-CT analysis with 3D reconstruction revealed osteophyte formation and impaired bone microarchitecture in the OA group (Fig. 5E, F, and S7). Treatment with EMM@HMs increased BV/TV and Tb.Th, while reducing the osteophyte volume and Tb.Sp.

**Fig. 6:**
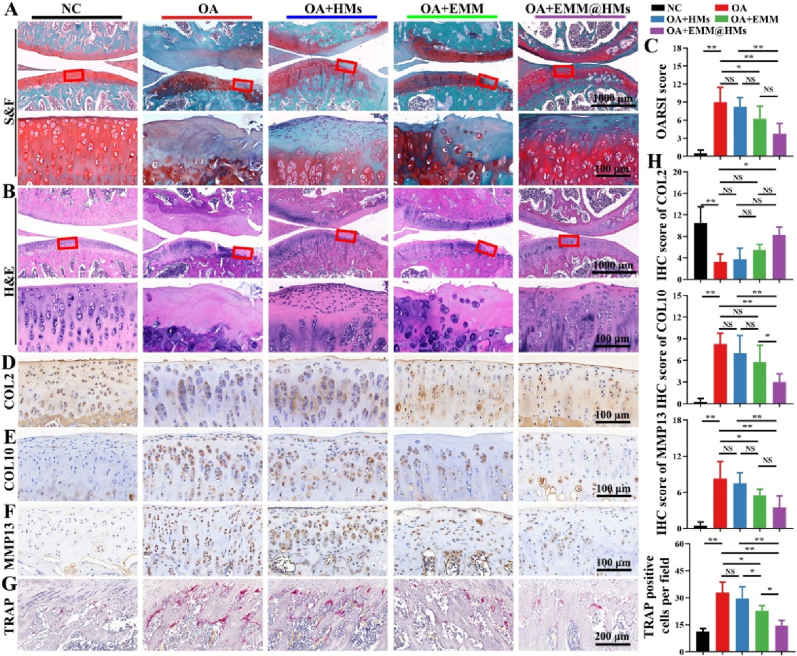
Histological and immunohistochemical evaluation of cartilage protection by EMM@HMs. (A) S&F staining and (B) H&E staining revealed severe cartilage degeneration in the OA group, and EMM@HMs preserved cartilage integrity. n = 4. (C) OARSI scoring showed significantly reduced cartilage damage in the EMM@HMs group. n = 4. (D) Immunohistochemical staining of COL2. n = 4. (E) COL10 and (F) MMP13 immunostaining demonstrated reduced cartilage hypertrophy and degradation with EMM@HMs treatment. n = 4. (G) TRAP staining indicated decreased bone resorption in the EMM@HMs group. n = 4. (H) Statistical analysis confirmed that EMM@HMs significantly protect cartilage and inhibit degeneration. n = 4. ∗p < 0.05, ∗∗p < 0.01, NS means not significant.

**Fig. 7:**
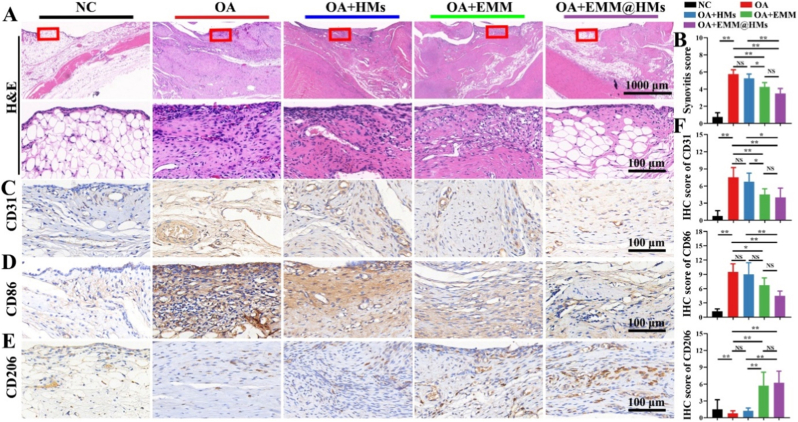
EMM@HMs alleviate synovial inflammation and regulate macrophage polarization in an OA rat model. (A) H&E staining of synovial tissue showed severe thickening and inflammatory cell infiltration in the OA group, which was reduced by EMM@HMs treatment. n = 4. (B) Synovitis scoring indicated synovial inflammation. n = 4. (C) Immunohistochemical staining for CD31 revealed elevated expression in the OA group, which was reduced by EMM@HM treatment. n = 4. (D) CD86 (M1 macrophage marker) and (E) CD206 (M2 macrophage marker) expression in synovial tissues. n = 4. (F) Statistical analysis of CD31, CD86, and CD206 immunohistochemical results. n = 4.

Histological and immunohistochemical analyses provided further insights into the protective effects of the EMM@HMs. S&F and H&E staining showed severe cartilage loss and matrix degradation in the OA group, whereas EMM@HMs preserved cartilage integrity and reduced OARSI scores (Fig. 6A−C). Immunohistochemical staining indicated that EMM@HMs enhanced the expression of the cartilage matrix marker COL2, while suppressing the expression of the cartilage degradation markers COL10 and MMP13 (Fig. 6D−F). TRAP staining revealed increased bone resorption activity in the OA group, which was attenuated by EMM@HMs treatment (Fig. 6G). Statistical immunohistochemical data are shown in Fig. 6H. Analysis of the synovial tissue revealed thickened synovium with significant inflammatory cell infiltration in the OA group. H&E staining showed that the EMM@HMs treatment alleviated these pathological changes (Fig. 7A and B). Immunohistochemical staining for CD31, CD86, and CD206 confirmed these findings. The OA group exhibited elevated levels of CD31^−^and CD86-positive cells, indicative of enhanced angiogenesis and M1 macrophage polarization, whereas the number of CD206-positive cells, representing M2 macrophages, was significantly reduced. EMM@HMs treatment decreased the number of CD31^−^and CD86-positive cells, while increasing the number of CD206-positive cells, suggesting its role in reducing angiogenesis, suppressing inflammation, and restoring immune balance in the synovium (Fig. 7C−E). Statistical data from immunohistochemical analysis of the synovial tissue are presented in Fig. 7F. In conclusion, these findings demonstrate that EMM@HMs mitigate inflammatory responses, preserve cartilage integrity, inhibit bone resorption, and modulate the synovial immune balance, highlighting their therapeutic potential for OA management.

### 3.6 EMM@HMs modulate key molecular pathways in cartilage repair and chondrocyte differentiation

**Fig. 8:**
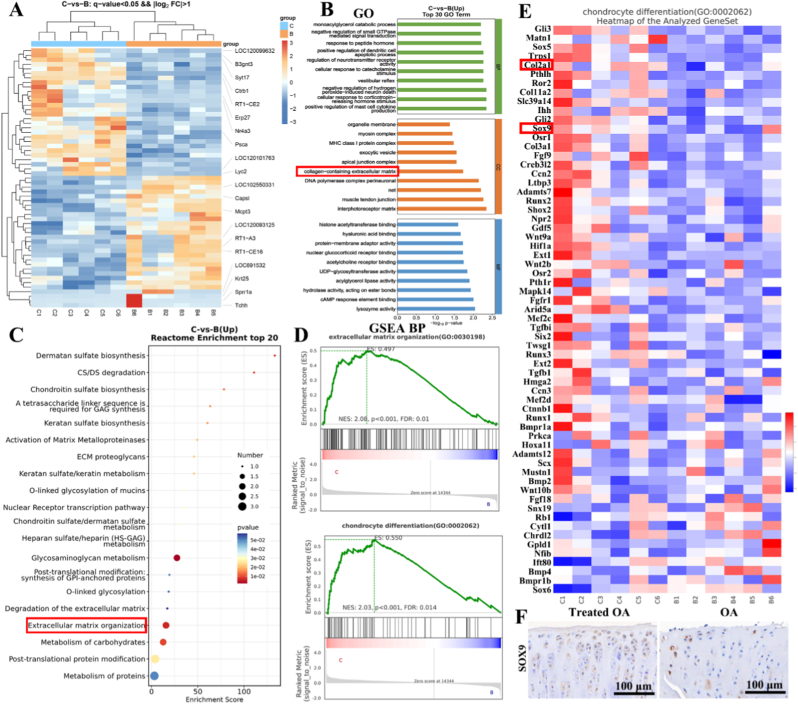
Transcriptomic analysis of EMM@HMs in OA. (A) A heatmap of DEGs revealed significant transcriptional changes associated with cartilage repair and ECM remodeling in the EMM@HMs-treated group compared to untreated OA samples. n = 6. (B) GO enrichment analysis highlighted processes of ECM organization and chondrocyte differentiation enriched in the EMM@HMs-treated group. (C) Reactome pathway analysis identified key pathways. (D) GSEA demonstrated significant enrichment of pathways. (E) Heatmap of genes involved in chondrocyte differentiation, including COL2A1 and SOX9. (F) Immunohistochemical staining of SOX9 expression in the EMM@HMs-treated group, indicating enhanced chondrocyte differentiation and cartilage repair. n = 4.

We conducted transcriptomic and bioinformatics analyses to explore the molecular mechanisms of EMM@HMs in OA treatment. Groups A, B, and C represent NC, OA, and EMM@HM-treated OA groups, respectively. The gene expression radar plot illustrates the differences in gene expression patterns across the different sample groups (Fig. S8A). Statistical analysis of DEGs indicated that, compared to the OA group, the treated group upregulated 28 genes and downregulated 29 genes, reflecting the regulatory effects of EMM@HMs on gene expression (Fig. S8B). Venn diagram analysis revealed overlapping and unique DEGs across treatment groups (Fig. S8C). The DEGs heatmap showed significant transcriptional changes between the OA and EMM@HMs-treated OA groups (Fig. 8A). Fig. S8D provides a comprehensive overview of GO enrichment, highlighting the biological processes closely associated with cartilage repair, including ECM organization, collagen metabolism, and chondrocyte differentiation. Fig. 8B, C focus on the upregulated GO and Reactome pathways, emphasizing significant enhancements in key functions, such as ECM structural organization, collagen binding, and chondrocyte differentiation. Together, these figures indicate that the EMM@HMs promote cartilage repair by upregulating molecular pathways related to ECM remodeling and chondrocyte function, providing evidence of the molecular mechanisms underlying their therapeutic effects on OA.

The GSEA in Fig. 8D reveals significant enrichment of key pathways related to cartilage repair, including collagen synthesis, cartilage regeneration, and ECM remodeling. Additionally, the GSEA analysis in Fig. S8E focuses on cellular components (CC) and molecular functions (MF), identifying significant enrichment in critical pathways, such as the collagen-containing extracellular matrix, extracellular matrix structural constituents, and collagen binding. These enriched pathways suggest that treatment promotes cartilage repair and regeneration by enhancing the structural stability of the ECM and collagen synthesis. The heat maps in Fig. 8E and S8F further concentrated on the GSEA biological process-enriched pathways, showing that EMM@HMs upregulated the expression of COL2A1 and SOX9 in the cartilage. Immunohistochemical analyses of COL2 and SOX9 further validated these transcriptomic findings, demonstrating that SOX9 protein expression was significantly higher in the EMM@HMs-treated groups (Figs. 6D and 8F).

### 3.7 EMM@HMs showed excellent biosafety in vivo

We analyzed the tissue morphology of the major organs, including the heart, liver, spleen, lungs, and kidneys, using H&E staining. The results revealed no significant tissue damage, inflammation, or pathological changes in any group (Fig. S9). Moreover, routine blood test results showed that stimulation with EMM@HMs did not cause significant changes in blood cell counts (Table S2). In addition, serum biochemical analysis demonstrated no obvious abnormalities in liver and kidney function indicators, further confirming the favorable biosafety of EMM@HMs (Fig. S10). These findings, together with the in vitro biocompatibility results, confirmed the favorable biosafety of EMM@HMs both in vitro and in vivo.

## 4 Discussion

OA is a common and complex degenerative joint disease characterized by cartilage degeneration, synovial inflammation, subchondral bone remodeling, and osteophyte formation. Although OA is often considered a disease rooted in mechanical wear and tears, increasing evidence suggests that its pathogenesis is driven by a highly dynamic process of low-grade chronic inflammation [28]. Unfortunately, currently no effective therapies are available that directly target the core nature of cartilage degeneration, and clinical interventions remain primarily focused on symptomatic relief and pain management.

Synovial abnormalities play a crucial role in the development of OA. As a heterogeneous connective tissue, the synovium is responsible not only for maintaining fluid balance and providing nutrients within the joint cavity but also acts as a key intermediary in OA pathology [29]. Under normal conditions, the synovium consists of an intimal layer with 2−3 layers of fibroblast-like synoviocytes (FLS) and resident macrophages, and a subintimal layer including fibroblasts, adipocytes, blood vessels, and a small number of immune cells. The intimal layer forms a porous barrier that isolates the joint cavity from the surrounding tissues [30]. Once OA progresses, leukocytes are continuously recruited to the subintimal layer and infiltrate the joint cavity through the intimal layer, aggravating synovial inflammation and cartilage degeneration [31].

Synovial macrophages originate from the bone marrow monocyte-macrophage lineage [32]. Functionally, they can differentiate into two diametrically opposing subtypes, proinflammatory M1 and anti-inflammatory M2 macrophages [33,34]. M1 macrophages highly express IL-1β, IL-6, TNF-α, and iNOS, thereby exacerbating synovial inflammation and promoting cartilage matrix degradation. By contrast, M2 macrophages secrete anti-inflammatory factors such as IL-10 and TGF-β1, inhibiting inflammation, reducing immune stress, and promoting tissue repair and regeneration [12,13,19,35]. In our study, we observed that TNF-α and IL-1β levels in OA synovial fluid increase with disease progression, and we confirmed that the EMM@HMs system can lower the levels of these two upstream proinflammatory factors, thereby comprehensively alleviating the inflammatory cascade.

TNF-α and IL-1β were selected as therapeutic targets because they occupy central and upstream positions in the OA inflammatory network. These cytokines not only initiate but also sustain inflammation by upregulating downstream mediators, such as IL-6, PGE2, MMPs, and ADAMTS5, creating a self-amplifying loop that drives cartilage degradation [36]. Although direct inhibition of MMPs and ADAMTS5 was once considered promising, clinical trials have demonstrated disappointing results owing to their regulation by upstream cytokines and their essential roles in normal tissue remodeling [[37], [38], [39]]. By contrast, targeting TNF-α and IL-1β can more effectively suppress the entire inflammatory cascade and reduce pathological enzyme expression, offering a broader disease-modifying effect [40,41]. Therefore, although this study mainly verified the neutralization of IL-1β and TNF-α, EMM@HMs may also indirectly modulate other inflammatory mediators by suppressing these upstream cytokine signals. For example, neutralization of TNF-α and IL-1β may reduce the downstream induction of IL-6, PGE2, MMPs, and ADAMTS5, thereby attenuating the broader inflammatory and catabolic microenvironment in OA. In addition, reduced TNF-α/IL-1β signaling may alleviate pro-inflammatory macrophage activation and facilitate a shift from M1-dominant inflammation toward M2-like immune regulation [42]. Nevertheless, these additional inflammatory factors were not systematically quantified in the present study, and broader cytokine profiling should be performed in future studies to further define the immunomodulatory effects of EMM@HMs.

The receptor for IL-1β belongs to the IL-1R family. The IL-1R family consists of structurally similar type I transmembrane proteins that typically have three domains: an extracellular region composed of three Ig-like domains responsible for ligand binding, a transmembrane domain, and an intracellular TIR domain [43]. The TIR domain plays a crucial role in initiating signal transduction. However, not all IL-1R family members can bind IL-1β. The receptors that can bind IL-1 include IL-1R1 and IL-1R2, both of which can bind IL-1α and IL-1β; their receptor antagonists include IL-1Ra and IL-1R3. Additionally, IL-1R4 is the receptor for IL-33, IL-1R5 is the ligand-binding chain for IL-18, and IL-1R6 is the receptor for three forms of IL-36, with IL-36Ra and IL-38 acting as receptor antagonists. Although both IL-1R1 and IL-1R2 can bind IL-1α and IL-1β, IL-1R2 has a higher specificity for IL-1β. Therefore, we selected IL-1R2 as the target for our study [[43], [44], [45]].

The receptor for TNF-α belongs to the TNFRs. Most TNFRs are type I transmembrane glycosylated proteins with an extracellular N-terminus and intracellular C-terminus, although some are type III transmembrane proteins. Structurally, TNFRs can be divided into three regions: extracellular domain (ECD), transmembrane domain (TMD), and intracellular domain (ICD). All TNFRs share a cysteine-rich domain (CRD) in the ECD region, which is responsible for the specificity and affinity of these receptors for their cognate ligands. Among them, the receptors for TNF-α are TNFR1 and TNFR2 [46]. Although both TNFR1 and TNFR2 can bind TNF-α, previous literature has reported that the TNF-α receptor antagonist soluble TNFR2 (sTNFR2) exhibits a higher affinity for TNF-α [[47], [48], [49], [50]]. By competitively binding, sTNFR2 inhibits TNF-α activity, thereby reducing the cascade reaction and suppressing inflammation. Therefore, we selected TNFR2 as the target for our study.

Despite the theoretical and preliminary experimental evidence supporting the efficacy of reducing IL-1β and TNF-α via IL1R2 and TNFR2, practical challenges remain. Achieving sustained, stable release and long-term presence in the joint cavity is a critical bottleneck in translating this strategy into clinical practice. To overcome this limitation, we selected microspheres as delivery vehicles and coated them with macrophage membranes. Compared with pure hydrogels or other carriers, microspheres excel in delivery efficiency, structural and functional maintenance, biocompatibility, multifunctionality, and mechanical stability. The cell membrane coating on the microspheres provides lubrication, decreases joint friction, and creates a favorable microenvironment for cartilage repair. Overall, microspheres surpass hydrogels and other materials in terms of delivery pathways, drug stability, sustained-release properties, and biological functional enhancements, making them an ideal choice for precision therapy in OA, intervertebral disc degeneration, and other chronic degenerative joint diseases [51,52].

Although EMM@HMs specifically absorb IL-1β and TNF-α, they indirectly promote M2 polarization by reducing the levels of these pro-inflammatory cytokines. Importantly, inhibition of IL-1β and TNF-α not only suppresses M1 activation but also creates a permissive environment for M2 polarization. By sequestering these cytokines, the EMM@HMs modulate the local immune microenvironment from a pro-inflammatory to an anti-inflammatory state, thereby promoting the differentiation of intermediate-state macrophages toward the M2 phenotype. This mechanism is supported by evidence from rheumatoid arthritis and inflammatory bowel disease. In these conditions, anti-TNF-α or anti-IL-1β therapies could reduce inflammation and promote a shift in macrophage phenotype from pro-inflammatory to anti-inflammatory. This phenotypic transition is accompanied by increased IL-10 secretion and reduced levels of TNF-α and IL-6 [53,54]. Our findings are consistent with this concept, suggesting that EMM@HMs regulate macrophage polarization via cytokine neutralization and modulation of the immune microenvironment.

Compared with other preclinical cartilage repair strategies, the major distinction of EMM@HMs is that this platform is designed to intervene in the upstream inflammatory microenvironment, rather than functioning solely as a passive trophic supplement or a cell-replacement strategy. In the present study, EMM@HMs acted as cytokine-scavenging “cellular sponges” by neutralizing IL-1β and TNF-α, facilitated M2 macrophage polarization, and concurrently attenuated cartilage degeneration while improving ECM-related markers. In addition, encapsulation of engineered macrophage membranes within HAMA microspheres improved their stability and intra-articular persistence relative to free EMM. Collectively, the combination of targeted cytokine sequestration, immunomodulatory activity, and prolonged local therapeutic availability represents a key feature that differentiates EMM@HMs from many existing OA biologics.

By comparison, stem cell-derived exosomes are widely recognized as promising cell-free therapeutics because they can attenuate synovial inflammation, protect chondrocytes, and support cartilage repair. However, their effects largely depend on cargo composition and paracrine signaling, and current reviews emphasize persistent translational challenges such as heterogeneity of source cells, batch-to-batch variability, purification, large-scale manufacturing, and standardization [55,56]. Thus, although exosome-based therapy is highly attractive, its mechanism is often indirect and its formulation consistency remains a major barrier.

PRP also has substantial preclinical and clinical interest because it can improve the joint milieu through growth factors and anti-inflammatory mediators, and several reviews support beneficial effects on pain and function. Nevertheless, PRP is inherently variable, since its composition depends on donor status and preparation protocol, and recent reviews note the lack of standardized preparation, dosing, and pharmacodynamic characterization [57]. Therefore, PRP is usually regarded as a broadly bioactive orthobiologic, but not a mechanism-defined platform for selective cytokine neutralization. In this regard, EMM@HMs may offer stronger mechanistic specificity by directly targeting two central OA cytokines, IL-1β and TNF-α.

With respect to M2 macrophage infusion, this strategy is conceptually close to our work because both aim to reprogram the inflammatory joint microenvironment. Prior studies have shown that engineered or “locked” M2a macrophages can suppress OA inflammation and promote regeneration. At the same time, those same studies highlight a major limitation of direct macrophage therapy: unmodified M2 macrophages may lose their anti-inflammatory phenotype and even switch toward M1 polarity in the OA milieu [58]. EMM@HMs potentially circumvent part of this problem by using engineered macrophage membranes rather than living cells, thereby reducing concerns related to cell survival, phenotype drift, and the unpredictability of adoptively transferred cells, while still preserving receptor-mediated cytokine interception.

From a clinical translation perspective, EMM@HMs should also be considered in relation to currently approved or clinically used OA drugs. Conventional pharmacological treatments, including topical/oral nonsteroidal anti-inflammatory drugs, intra-articular corticosteroids, acetaminophen, duloxetine, tramadol, topical capsaicin, and intra-articular hyaluronic acid products, mainly aim to relieve pain and improve symptoms rather than directly reverse cartilage degeneration or reshape the upstream inflammatory microenvironment [[59], [60], [61]]. In contrast, EMM@HMs are designed as a locally administered cytokine-neutralizing biomaterial system that targets IL-1β and TNF-α, two upstream inflammatory mediators in OA. Therefore, compared with conventional symptomatic drugs, EMM@HMs may offer potential translational advantages in local immune modulation, prolonged intra-articular retention, reduced systemic exposure, and combined anti-inflammatory/cartilage-protective effects. However, unlike approved OA drugs, EMM@HMs remain at the preclinical stage and require further validation before clinical translation. Key challenges include long-term safety, potential immunogenicity of engineered membrane components, scalable manufacturing, sterilization, batch-to-batch quality control, optimal dosing regimens, and regulatory classification. Thus, EMM@HMs should currently be regarded as a promising experimental strategy that may complement, rather than replace, existing approved OA therapies. Taken together, EMM@HMs may serve as a complementary experimental strategy by integrating cytokine neutralization, macrophage-oriented immune regulation, prolonged local retention, and cartilage-protective effects.

Notably, the construction of cartilage organoids represents a major advancement in regenerative medicine and tissue engineering. Cartilage organoids, formed by tissue engineering techniques in vitro, not only emulate the morphology and function of native cartilage but also offer new experimental and translational platforms for studying OA pathogenesis, drug screening, and clinical applications [22]. In this study, we used ATDC5 cells for the first time to construct homogeneous, spherical cartilage organoids that more closely resemble native cartilage microstructures than ATDC5 cells cultured under traditional two-dimensional conditions. Further findings indicate that EMM@HMs can absorb and eliminate TNF-α and IL-1β, inhibiting cartilage degradation and chondrocyte hypertrophy, thus laying the groundwork for integrating organoid technology with our strategy to achieve more efficient cartilage regeneration and repair in the future.

Nevertheless, we acknowledge that the current cartilage organoid model cannot fully reproduce the biomechanical properties of native articular cartilage. The Young's modulus of our cartilage organoids was approximately 400 kPa, which overlaps only with the lower range of native cartilage stiffness. This mechanical difference may limit their ability to simulate mechanically driven OA progression, such as changes caused by long-term compression, shear stress, or abnormal joint loading. However, the primary purpose of this model was to evaluate inflammation-induced cartilage degeneration and the protective effects of EMM@HMs in a three-dimensional cartilage-like system. Since OA-like changes were mainly induced by M1 macrophage-conditioned medium, the relatively low modulus is unlikely to fundamentally affect our conclusion regarding the anti-inflammatory and cartilage-protective effects of EMM@HMs. In addition, the therapeutic efficacy was further validated in the rat OA model, which better preserves the native joint mechanical environment.

Cartilage organoid research is aimed at achieving breakthroughs in multiple areas. By integrating gene editing, epigenetic regulation, synthetic biology tools, and high-throughput drug screening technologies, researchers can fine-tune the cellular composition, matrix constitution, and mechanical properties of organoids to create higher-fidelity models. Building on these foundations, incorporating 3D bioprinting, microfluidic arrays, and intelligent, programmable biomaterials can enable the customized and scalable production of organoids tailored to specific disease states and patient needs. In particular, the incorporation of dynamic compression, shear stimulation, zonal structure design, and longer maturation protocols may help generate cartilage organoids with mechanical characteristics closer to those of native cartilage. Furthermore, combining organoid technology with in vivo transplantation and real-time monitoring methods (such as high-resolution imaging and labeling/tracking techniques) allows the dynamic evaluation of tissue regeneration after transplantation. This helps clinicians make timely adjustments to treatment strategies, thereby improving treatment success rates and patients’ quality of life [21].

## 5 Conclusion

The EMM@HMs delivery strategy proposed in this study provides a new approach to the prevention and treatment of OA by directly targeting upstream inflammatory factors, TNF-α and IL-1β. With the sustained-release and precise delivery capabilities of the microsphere carrier, along with the strategic use of the TNFR2 and IL1R2 signaling pathways, this method demonstrates remarkable potential for alleviating inflammation and promoting cartilage repair. Introducing organoid technology into OA research will establish a solid foundation for bridging the gap between laboratory findings and clinical practice. As cross-disciplinary integration and technological convergence continue to deepen, the rapid advancement of cartilage organoids and related precision therapies promises to open a new chapter in regenerative medicine and tissue engineering, guiding the way toward precise, long-lasting, and safe interventions for OA and other degenerative joint diseases.

## CRediT authorship contribution statement

**Chenggong Ma:** Data curation, Methodology. **Zhisheng Xiao:** Methodology, Software. **Yetian Ma:** Data curation, Methodology, Software. **Wenwei Jiang:** Methodology, Writing – original draft. **Yufan Qian:** Investigation, Project administration. **Zicheng Deng:** Resources, Visualization, Writing – original draft. **Jiong Jiong Guo:** Conceptualization, Resources, Supervision. **Qian Chen:** Supervision, Validation, Writing – review & editing. **Feng Zhou:** Conceptualization, Writing – review & editing.

## Declaration of competing interest

The authors have no competing interests to declare in relation to this article.

## Data Availability

Data will be made available on request.
