# Supplementary material for: Biomimetic cellular sponges for neutralizing inflammatory cytokines in osteoarthritic joints
Source: Mater Today Bio. 2026 Jun 11;39:103347. doi: 10.1016/j.mtbio.2026.103347 (PMC13276572; doi:10.1016/j.mtbio.2026.103347)
Supplement: Multimedia component 1 [file mmc1.docx]

Biomimetic cellular sponges for neutralizing inflammatory cytokines in osteoarthritic joints

Chenggong Ma^a,1^, Zhisheng Xiao^b,1^, Yetian Ma^a,1^, Wenwei Jiang^c,1^, Yufan Qian^a^, Zicheng Deng^a^, Jiong Jiong Guo^a,*^, Qian Chen^b,*^, Feng Zhou^a,*^

^a^ Department of Orthopaedic Surgery, The First Affiliated Hospital of Soochow University, Orthopedic Institute, Suzhou Medical College, Soochow University, Suzhou, Jiangsu, 215000, P. R. China.

^b^ Institute of Functional Nano and Soft Materials (FUNSOM), Jiangsu Key Laboratory for Carbon-Based Functional Materials and Devices, Soochow University, Suzhou, Jiangsu, 215123, P. R. China.

^c^ Department of Orthopaedic Surgery, Kunshan Hospital of Chinese Medicine, Affiliated Hospital of Yangzhou University, Jiangsu, 215300, P. R. China.

^1^ These authors contributed equally to this work.

^*^ Corresponding authors: zhoufeng16@suda.edu.cn, chenqian@suda.edu.cn, drjjguo@163.com.


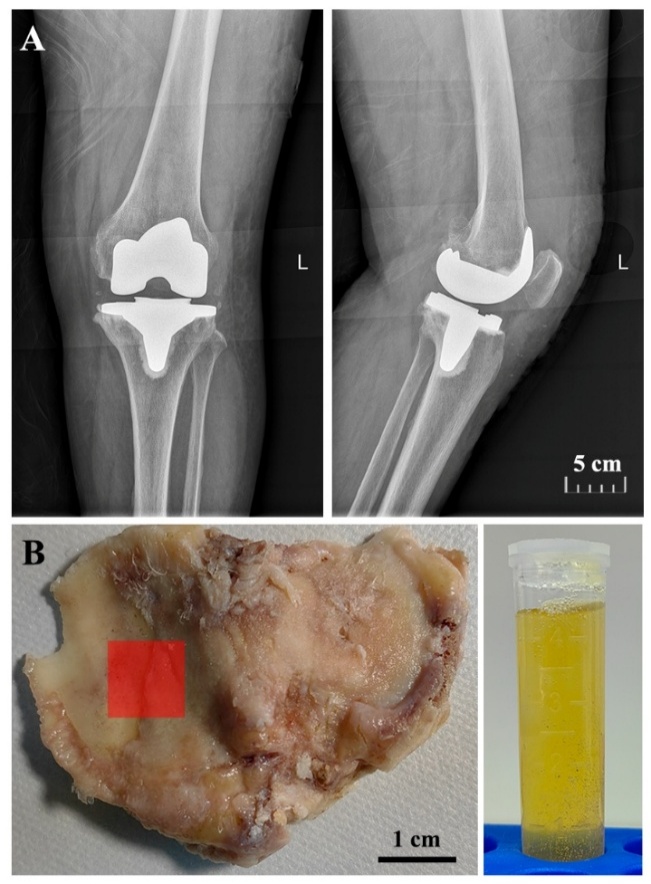


Figure S1. (A) Postoperative radiographs of the knee joint following TKA. (B) Articular cartilage and synovial fluid samples collected during surgery.


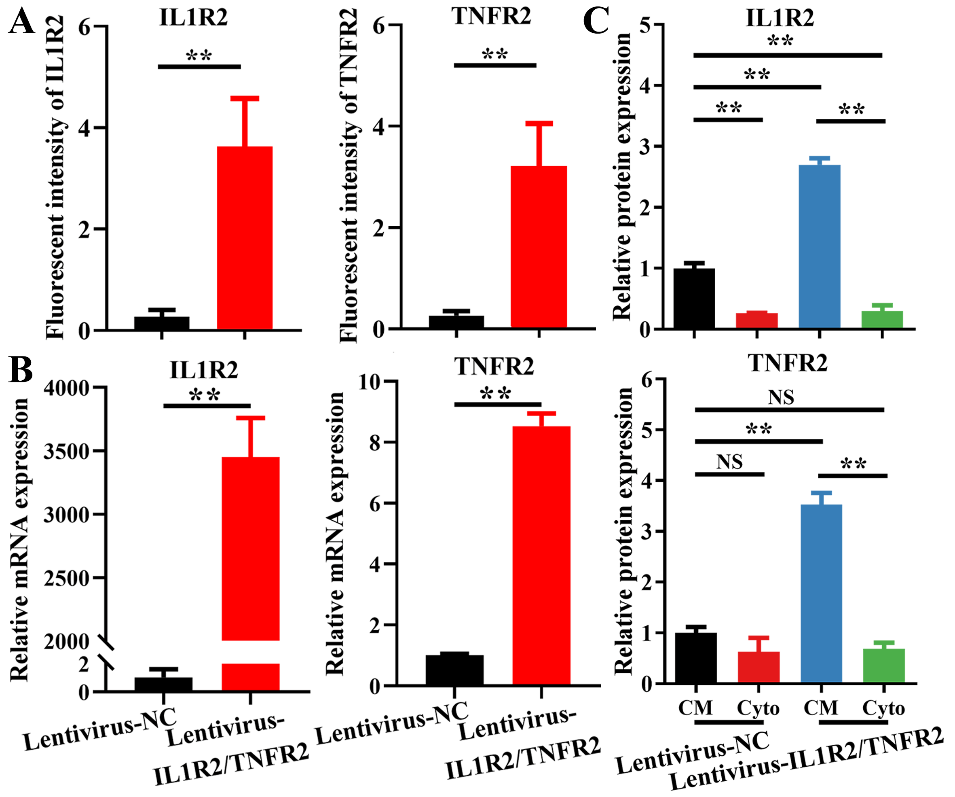


Figure S2. Validation of IL1R2 and TNFR2 overexpression after lentiviral transfection. (A) Quantitative analysis of the immunofluorescence intensity of IL1R2 and TNFR2 in RAW264.7 after lentiviral transfection. n=4. (B) qPCR analysis showing the mRNA expression levels of IL1R2 and TNFR2 after lentiviral transfection. n=3. (C) Analysis of western blot showing IL1R2 and TNFR2 expression in the cell membrane (CM) and cytoplasm (Cyto) of RAW264.7 after transfection. n=3. ** p<0.01, NS means not significant.


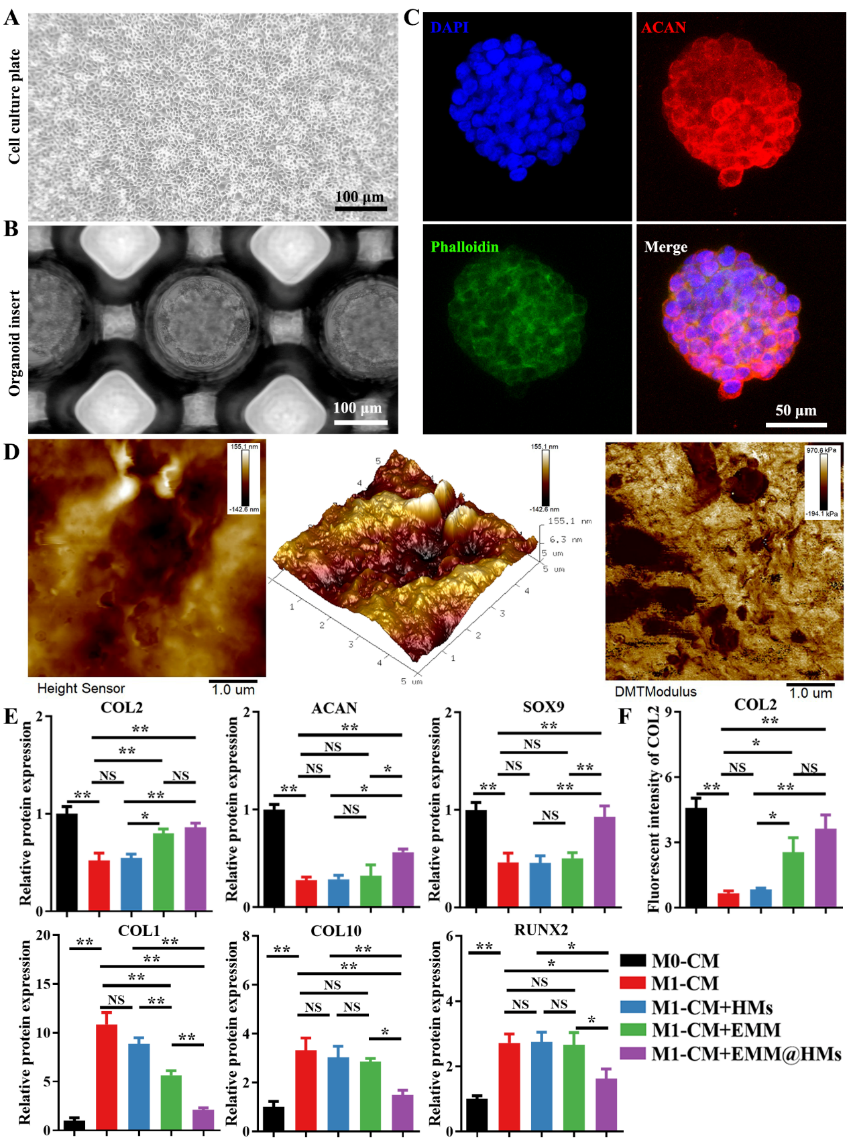


Figure S3. Morphological and phenotypic characterization of cartilage organoids. (A) Bright-field image of ATDC5 cells cultured in conventional plates, showing typical monolayer morphology. (B) Bright-field image of ATDC5 cells cultured in organoid inserts, showing spherical aggregates. (C) Immunofluorescence staining of cartilage organoids for ACAN. (D) Representative AFM images of cartilage organoids, including height map, 3D surface topography, and Young’s modulus map. (E) Quantitative analysis of western blot results. (F) Quantitative analysis of COL2 immunofluorescence intensity in cartilage organoids. n=3. * p<0.05, ** p<0.01, NS means not significant.


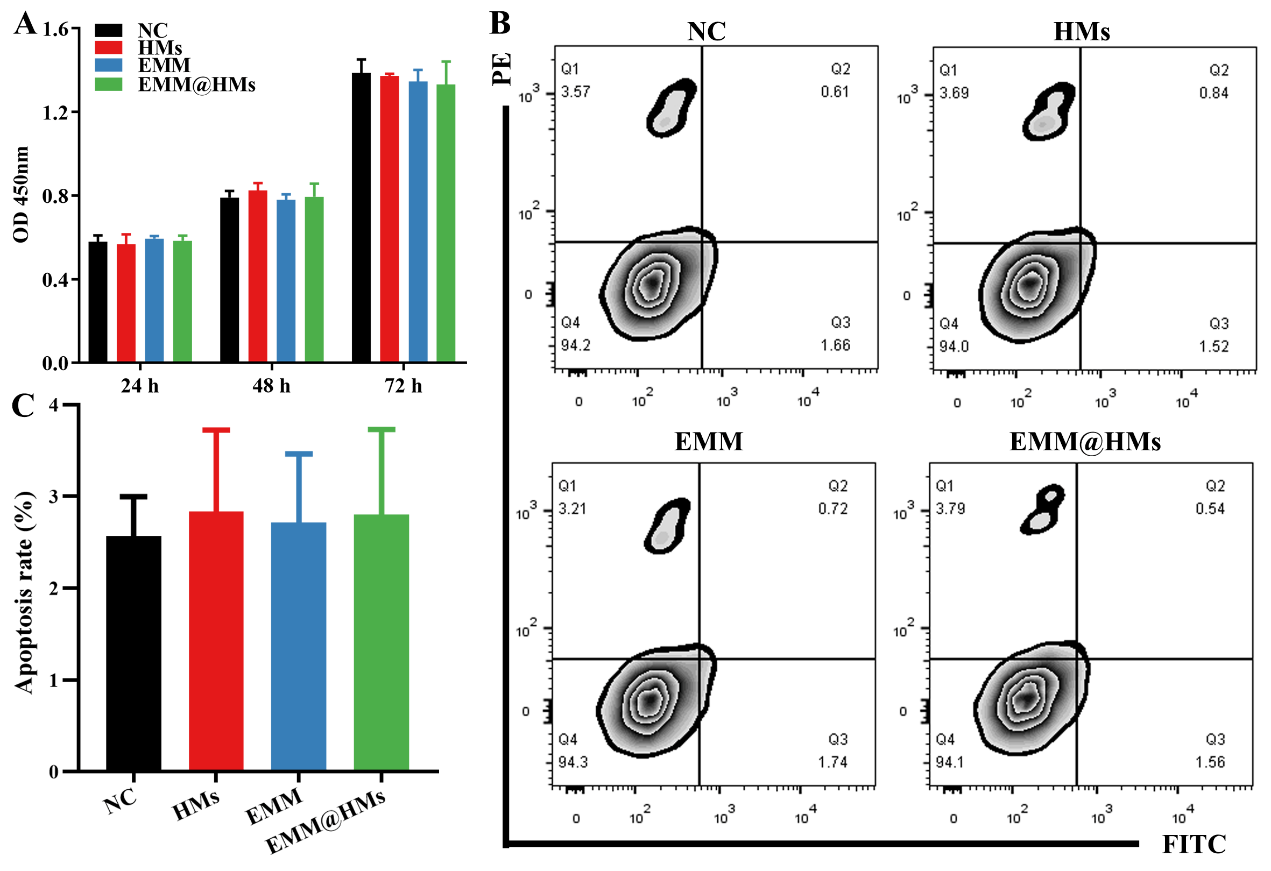


Figure S4. In vitro biocompatibility evaluation of HMs, EMM, and EMM@HMs. (A) CCK-8 assay demonstrated cell viability among NC, HMs, EMM, and EMM@HMs groups. n=3. (B) Flow cytometry analysis showed apoptosis levels. (C) Quantitative analysis of flow cytometry confirmed the biosafety of EMM@HMs. n=3.


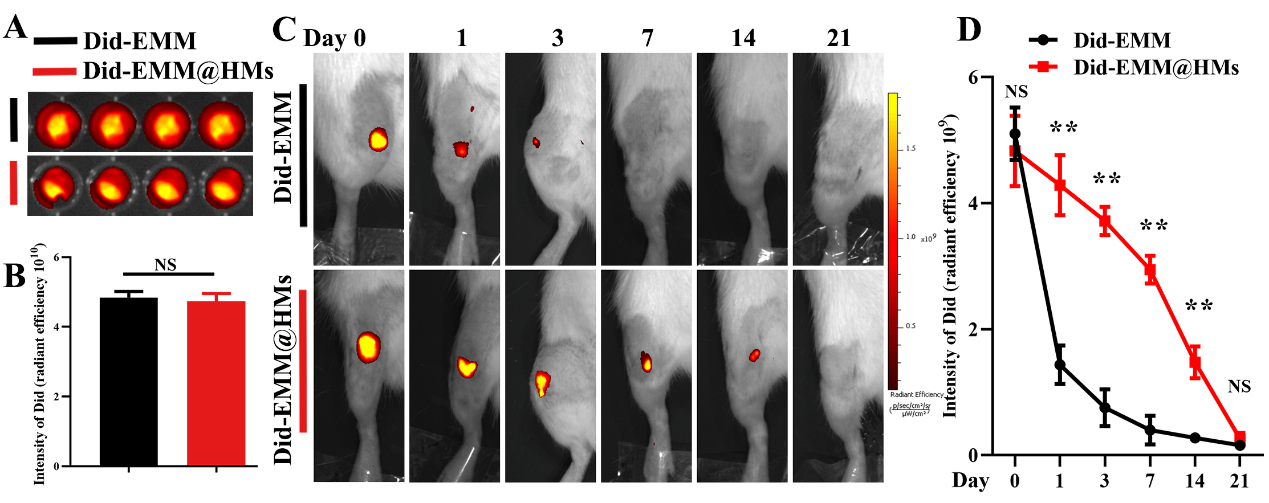


Figure S5. Prolonged intra-articular retention of EMM@HMs. (A) In vivo fluorescence images of Did-EMM and Did-EMM@HMs detected by IVIS. (B) Quantitative analysis of fluorescence intensity for Did-EMM and Did-EMM@HMs. n=4. (C) Fluorescence imaging of rat knee joints after intra-articular injection at different time points. (D) Quantitative analysis of fluorescence intensity in the injected joints. n=4. ** p<0.01, NS means not significant.


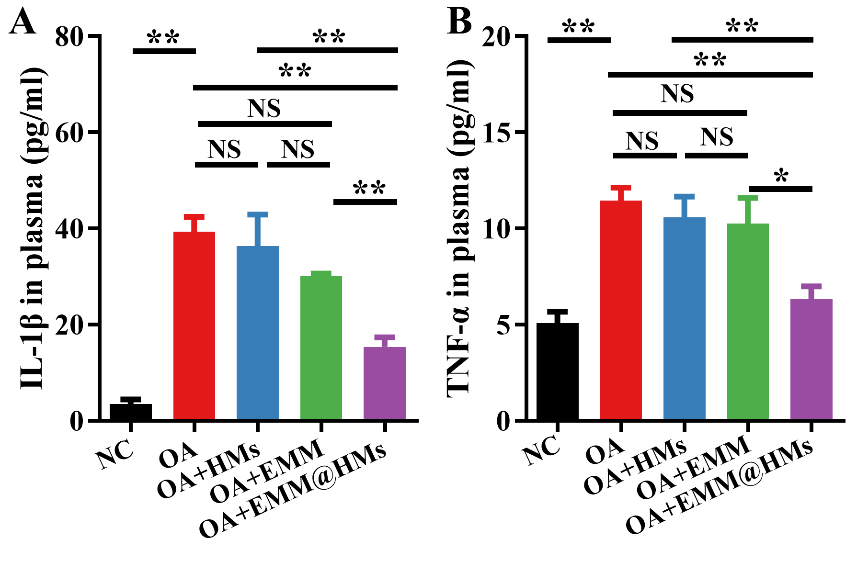


Figure S6. Plasma levels of IL-1β and TNF-α. (A) IL-1β and (B) TNF-αlevels were elevated in the OA group and reduced by EMM@HMs treatment. n=4. * p<0.05, ** p<0.01, NS means not significant.


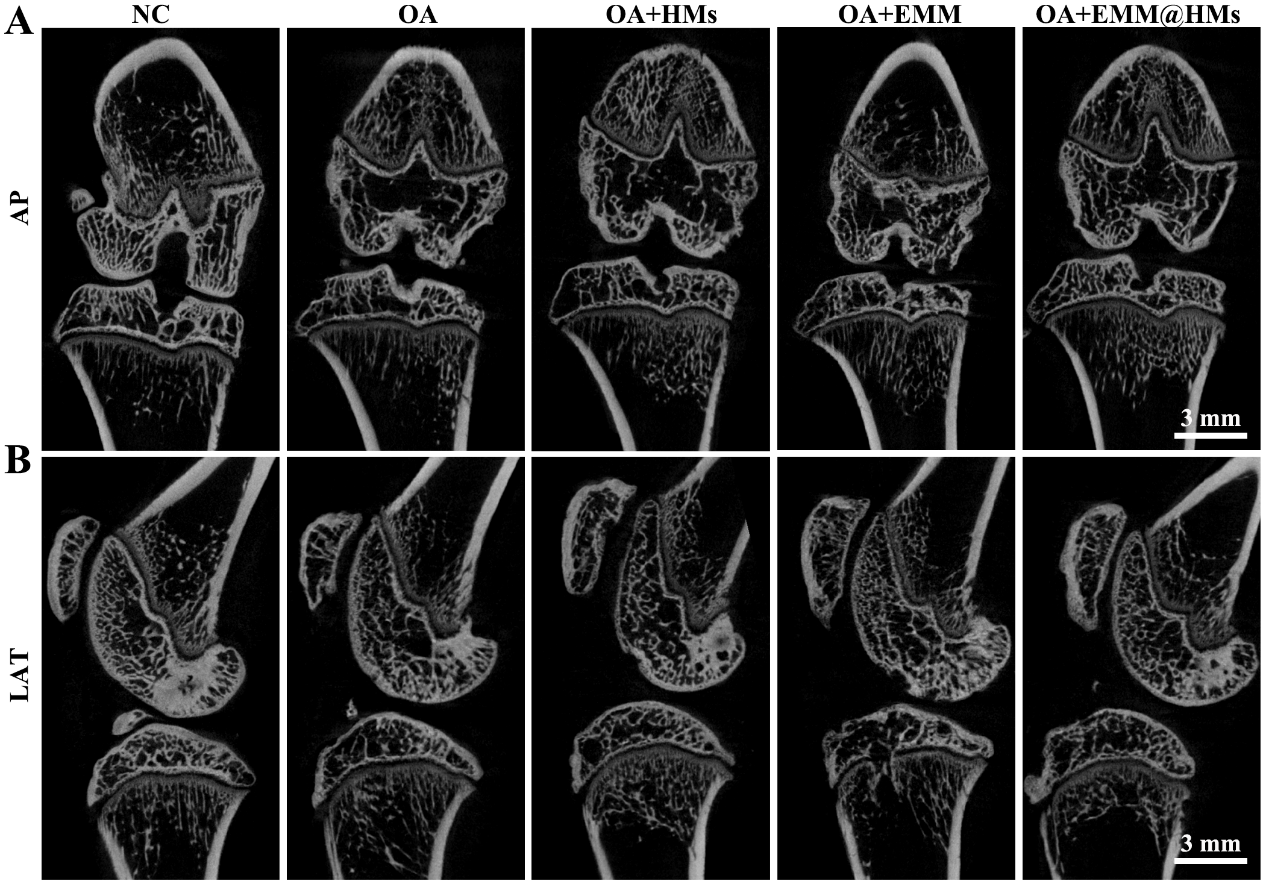


Figure S7. Micro-CT 2D reconstruction of knee joints under different treatments. (A) Anteroposterior and (B) lateral views of knee joints in various groups. n=4.


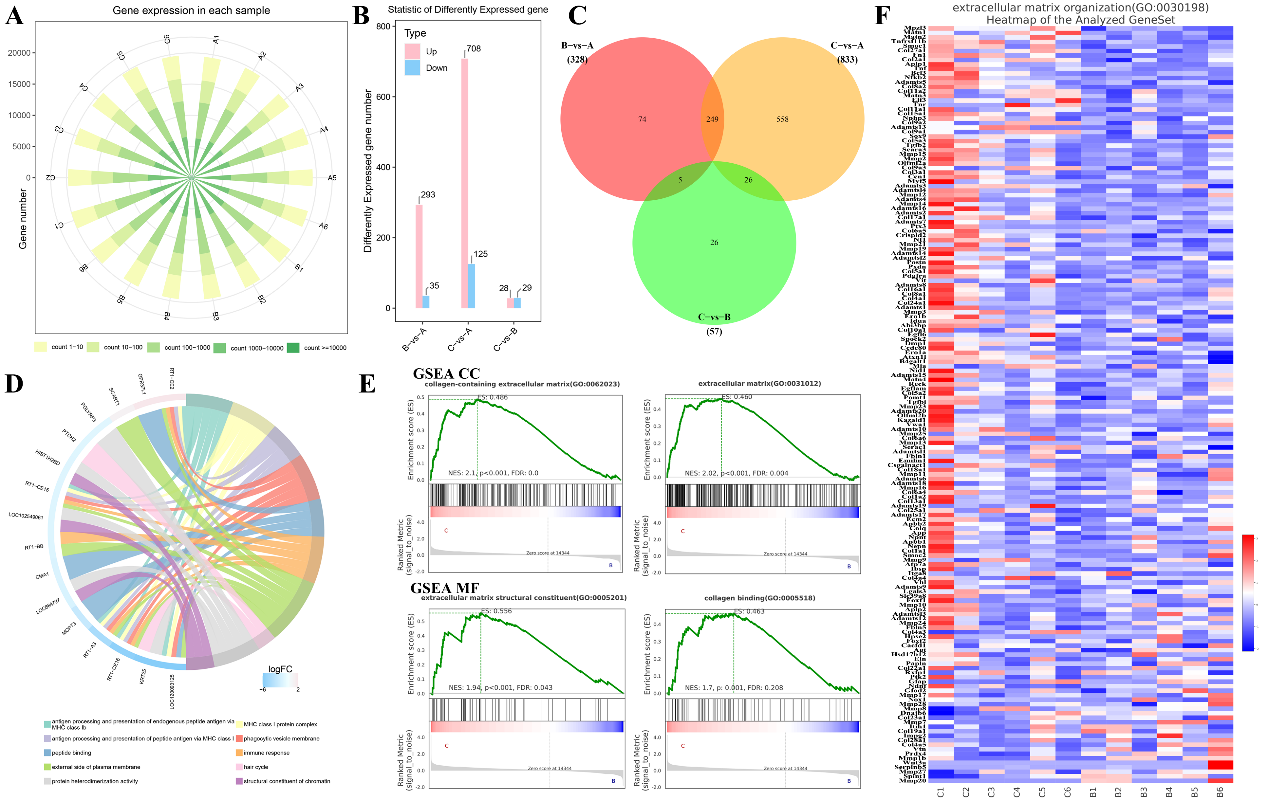


Figure S8. Transcriptomic analysis of differentially expressed genes and pathways modulated by treatments. (A) Radial plot showed gene expression distribution across samples. (B) Bar chart of DEGs highlighting upregulated and downregulated genes. (C) Venn diagram showed overlapping and unique DEGs among the comparisons. (D) Chord diagram showed significant DEGs to biological processes. (E) GSEA results for CC and MF highlighted enriched pathways. (F) Heatmap of genes involved in ECM organization showed distinct expression patterns. n=6.


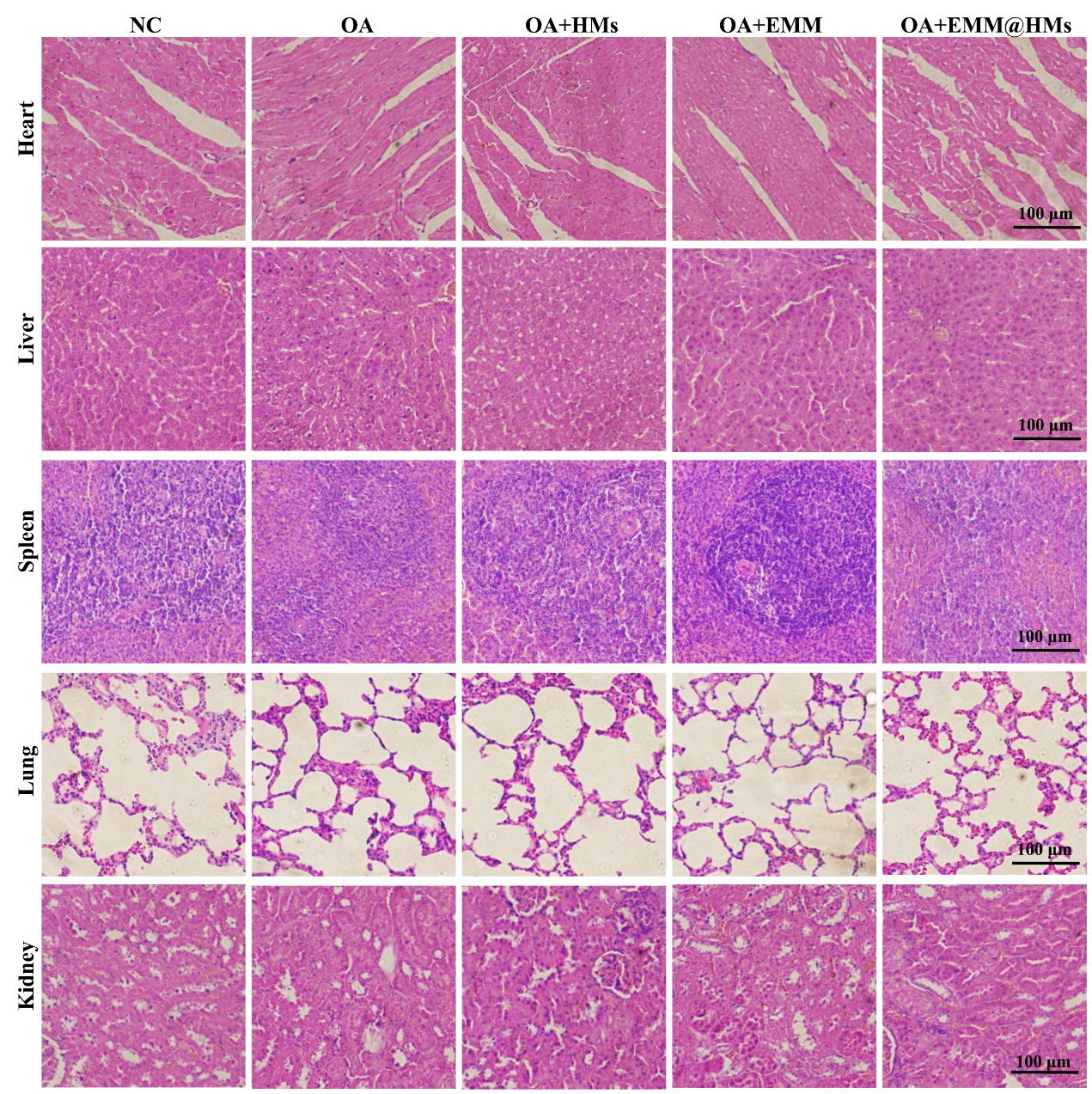


Figure S9. In vivo biosafety evaluation of EMM@HMs. Representative H&E staining sections of major organs including heart, liver, spleen, lung, and kidney from different treatment groups. n=4.


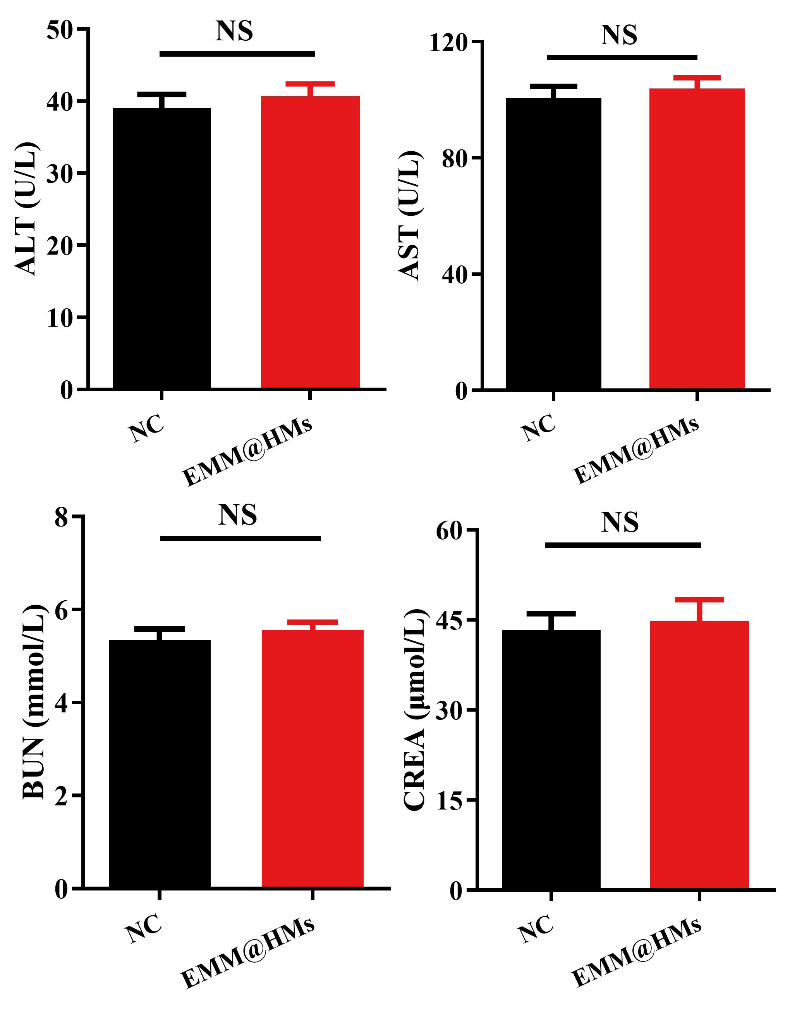


Figure S10. Serum biochemical analysis of liver and kidney function in rats treated with or without EMM@HMs. n=6.

Table S1. Gene primers in qPCR analysis

| β-actin-F | 5’-GGCTGTATTCCCCTCCATCG-3’ |
| --- | --- |
| β-actin-R | 5’-CCAGTTGGTAACAATGCCATGT-3’ |
| IL1R2-F | 5’- GTTTCTGCTTTCACCACTCCA-3’ |
| IL1R2-R | 5’- GAGTCCAATTTACTCCAGGTCAG-3’ |
| TNFR2-F | 5’- ACACCCTACAAACCGGAACC-3’ |
| TNFR2-R | 5’- AGCCTTCCTGTCATAGTATTCCT-3’ |
| COL2A1-F | 5’-CACCCTCAA ATCCCTCAA CAATCA G-3’ |
| COL2A1-R | 5’-TGTCTTTCGTCTTGCTGGTCCACC-3’ |
| ACAN-F | 5ʹ-CAGTGCGATGCAGGCTGGCT-3ʹ |
| ACAN-R | 5ʹ-CCTCCGGCACTCGTTGGCTG-3ʹ |
| SOX9-F | 5’-TACCTACGGCATCAGCAGCTC-3’ |
| SOX9-R | 5’-TTGCCTTCACGTGGCTTTAAG-3’ |
| COL1A1-F | 5’-GCTCCTCTTAGGGGCCACT-3’ |
| COL1A1-R | 5’-CCACGTCTCACCATTGGGG-3’ |
| COL10A1-F | 5’-AAAGCTTACCCAGCAGTAGG-3’ |
| COL10A1-R | 5’-ACGTACTCAGAGGAGTAGAG -3’ |
| RUNX-2-F | 5’-TCCCCGGGA ACCAAGAAGGCA-3’ |
| RUNX-2-R | 5’-AGGGAGGGCCGTGGGTTCTG-3’ |

Table S2. Blood analysis of rats treated with or without EMM@HMs. n=6

|  | NC | EMM@HMs | p value (t test) |
| --- | --- | --- | --- |
| WBC (10^9^/L) | 6.600±2.524 | 7.000±2.601 | 0.8101 |
| Lymph (10^9^/L) | 4.933±1.955 | 5.083±2.023 | 0.9075 |
| Mon (10^9^/L) | 0.167±0.094 | 0.183±0.090 | 0.7805 |
| Gran (10^9^/L) | 1.500±0.619 | 1.733±0.797 | 0.6165 |
| Lymph (%) | 74.667±4.594 | 72.283±6.557 | 0.5207 |
| Mon (%) | 2.700±0.693 | 2.900±0.835 | 0.6888 |
| Gran (%) | 22.633±4.129 | 24.817±6.087 | 0.5219 |
| RBC (10^12^/L) | 6.207±2.767 | 5.423±1.144 | 0.5714 |
| HGB (g/L) | 113.167±13.234 | 109.000±9.950 | 0.5860 |
| HCT (%) | 34.467±16.115 | 29.317±6.407 | 0.5217 |
| MCV (fl) | 55.200±2.550 | 54.033±1.362 | 0.3881 |
| MCH (pg) | 17.333±3.593 | 18.533±0.373 | 0.4747 |
| MCHC (g/L) | 316.000 ±66.290 | 344.000±5.066 | 0.3685 |
| RDW (%) | 13.467±1.579 | 12.833±1.039 | 0.4709 |
| PLT (10^9^/L) | 674.333±269.959 | 731.500±228.201 | 0.7252 |
| MPV (fl) | 8.417±0.518 | 7.033±1.310 | 0.0528 |
| PDW | 17.267±0.553 | 16.783±0.570 | 0.2033 |
| PCT (%) | 0.516±0.190 | 0.471±0.166 | 0.7279 |
